# Recent progress and future prospects of the early solar system chronology

**DOI:** 10.1093/nsr/nwaf281

**Published:** 2025-07-11

**Authors:** Yuri Amelin, Qing-Zhu Yin

**Affiliations:** State Key Laboratory of Deep Earth Processes and Resources, Guangzhou Institute of Geochemistry CAS, Guangzhou 510640, China; Department of Earth and Planetary Sciences, University of California, Davis, Davis, CA 95616, USA

**Keywords:** cosmochronology, early solar system, isotope dating, meteorite, extraterrestrial materials

## Abstract

We review the developments in isotope chronology of the oldest extraterrestrial materials achieved in the last 10–15 years, with emphasis on high precision U–Pb and extinct radionuclide dating, and on application of these methods to the materials that formed in the first 7 million years after formation of proto-Sun, the time during which the gas and dust in the protoplanetary disc accreted or dissipated, and planetesimals and protoplanets formed. The analytical precision of isotopic dates now allows resolving events that occurred within 100 000–300 000 years of each other. The main challenges currently faced by isotope cosmochronology are matching the achieved precision with a similar level of accuracy, adapting to the growing evidence of complex isotope heterogeneity of the protoplanetary disc, extracting ages of individual events from complex rocks, and responding to growing quantity, quality and diversity of recently discovered meteorites and samples returned by space missions.

## INTRODUCTION

In the last 70 years, isotopic dating of extraterrestrial materials, also called cosmochronology, went a long way from a method that allowed the centuries-long debate about the age of our planet [1] to be settled, to a versatile research toolbox that is indispensable for understanding formation of the solar system. This toolbox includes a number of chronometers based on decay of extant and extinct radionuclides with a variety of nuclear properties and chemical affinities. Together, these chronometers allow precise dating of processes that produced and transformed extraterrestrial rocks: condensation; evaporation; metal–silicate separation; melt crystallisation; metamorphism; metasomatism; and impacts.

The progress of cosmochronology since its inception and its status in the mid-2000s was summarised in 2009 in the special issue of *Geochimica et Cosmochimica Acta* [2], based on the papers presented at the 2007 Workshop on Chronology of Meteorites and the Early Solar System in Kauai, HI, USA, and honouring the contributions of the pioneers of the solar system chronology (C.J. Allègre, G.W. Lugmair, L.E. Nyquist, D.A. Papanastassiou, and G.J. Wasserburg). The status of this field about 15 years ago is also reflected in the chapters by Davis and McKeegan [3] and Wadhwa [4] in the second edition of *Treatise on Geochemistry*. The last 10–15 years following these publications have been eventful years in early solar system (ESS) research, which has brought new methods, new data and new ideas, and changed the landscape of research in the ESS chronology.

This paper is not intended to cover all aspects of the ESS chronology in full detail. It is complementary to the recent reviews [5–7]. In this paper, we review new developments in the context of problems revealed by past research, and the gaps in knowledge that need to be filled by future studies. We focus exclusively on the time measured with isotope chronometers, including the time intervals based on decay of relatively short-lived radionuclides that are currently extinct, and the ages based on decay of extant radionuclides of ^238^U and ^235^U to radiogenic ^206^Pb and ^207^Pb. We review applications of age data acquired using these chronometers to understand the history of planetary bodies through materials that formed in the accreting solar protoplanetary disc and preserved within asteroids.

## THE PROGRESS IN ANALYTICAL PRECISION AND ACCURACY

The effectiveness of geochronology as a problem-solving tool hinges on the precision of isotopic dating, which constrains our ability to determine the timing of past events. We first discuss the progress, and related problems, of U–Pb dating in the ^207^Pb*/^206^Pb* form (asterisk denotes radiogenic Pb)—the widely used system for dating extraterrestrial rocks and minerals. We then turn to other isotopic chronometers, as well as general problems applicable to all chronometer systems.

### Precision of Pb isotopic ages

**Figure 1.**
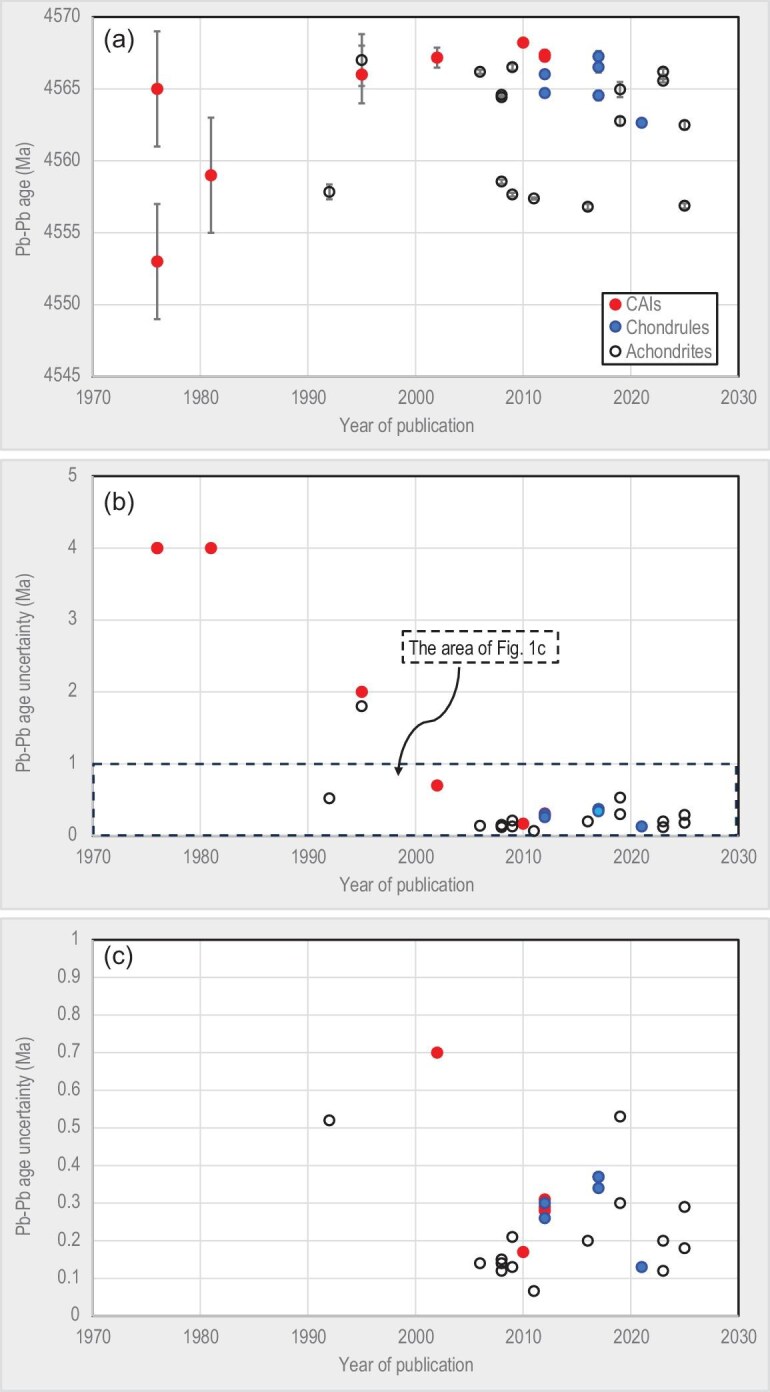
(a) Pb isotopic ages of CAIs, chondrules and achondrites, published between the mid-1970s and the end of 2024. The ages and their uncertainties are shown as reported in the original publications. Only the data based on highly radiogenic Pb isotope analyses, and determined for materials with minimal contamination and disturbance, are included in the plot. (b, c) Uncertainties of the ages shown in (a).

The precision of Pb isotopic dates of meteorites and their components have changed greatly since the first studies of Allende calcium–aluminium-rich inclusions (CAIs) in the mid-1970s (Fig. 1). Most data are obtained with isotope dilution thermal ionisation mass spectrometry (ID-TIMS), but some were measured with multicollector inductively coupled plasma mass spectrometry (MC-ICPMS). Only the data based on highly radiogenic Pb–isotope analyses, and determined for materials free from significant contamination and disturbance, are included in the plot, hence the precision of the dates approximates analytical state-of-the-art of their time. The ages determined in the 1970s and 1980s have uncertainties of about 4 Ma. In the 1990s and early 2000s, the uncertainties of the best age determinations were between 0.5 and 2.0 Ma. The second half of 2000s saw a dramatic drop of age uncertainties to ∼0.1–0.3 Ma due to a combined effect of analytical novelties introduced at that time (e.g. [8–11]): ^202^Pb–^205^Pb spike used to correct for within-run fractionation drift; lower blanks; higher sensitivity due to use of more efficient emitters; and more sophisticated partial dissolution procedures that helped to minimise uncertainty of non-radiogenic Pb subtraction.

Despite further analytical developments, such as new electrometer amplifiers with reduced baseline noise, which improve signal-to-noise ratio in analyses of medium to small ion beams typical for Pb isotopic dating [12,13], the best reported precision of Pb isotopic ages has remained unchanged since the second half of the 2000s.

### ^238^U/^235^U variations and the accuracy of Pb isotopic ages

While the state-of-the-art precision of Pb isotopic dating has remained unchanged in the last ∼15 years, the accuracy has improved substantially. The most important change has been consideration of variability of the ^238^U/^235^U ratio. In the earlier days of cosmochronology, uniformity of ^238^U/^235^U at the ∼0.3%–2.0% level in Allende CAIs and meteoritic phosphates [14] was taken as evidence that the ^238^U/^235^U ratio in extraterrestrial materials is constant. This notion was reinforced by more precise MC-ICPMS U isotopic data for various chondrites, achondrites and their components [15,16] obtained in a search for live ^247^Cm in the ESS. The discovery of ^238^U/^235^U variability in terrestrial rocks [17,18] stimulated further searches for U isotopic variations in meteoritic materials, which led to the discovery of well-resolved ^238^U/^235^U variations in Allende CAIs [19], reaching up to 0.34%. Such deviations required adjustment to the Pb–Pb ages of up to 4.9 Ma, far greater than the uncertainty of meteorite Pb isotopic dating.

The importance of ^238^U/^235^U variability was immediately recognised, and the practice of using directly measured ^238^U/^235^U ratios in meteorite dating was established [11,20,21]. Furthermore, for sufficiently homogeneous materials, e.g. achondrites, it became possible to apply precise ^238^U/^235^U ratios obtained in dedicated studies [22–24] to Pb isotopic dates that were published before the U isotope variability was discovered.

**Figure 2.**
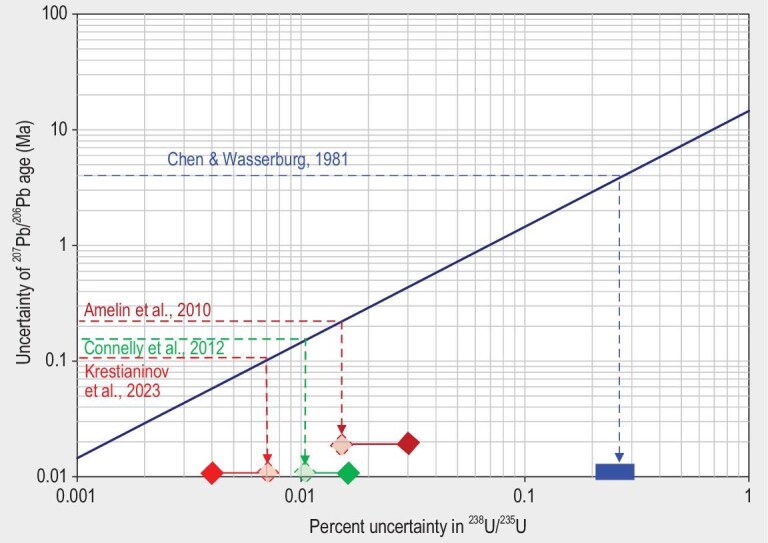
Uncertainty of ^238^U/^235^U required for correction of Pb–Pb ages. The horizontal lines cross the *y*-axis of uncertainties of the ^207^Pb/^206^Pb ages at the values corresponding to the uncertainty of ^207^Pb*/^206^Pb* dates calculated directly from isochron regressions or model dates, without considering the uncertainty of the ^238^U/^235^U ratios (±4 Ma for Allende CAIs in [159], ±0.21 Ma for Allende CAI SJ101 in [20], ±0.15 Ma for Efremovka CAI 22E in [11], calculated from the data in their Table S4, and ±0.12 Ma for achondrite Erg Chech 002 in [25]). The diagonal line is a locus of equal contributions from ^207^Pb*/^206^Pb* and ^238^U/^235^U uncertainties to the total uncertainty of the age. The projections by vertical arrows to the *x*-axis (marked with semi-transparent symbols) show the values of uncertainty of the ^238^U/^235^U ratios that are required to match the uncertainty of the ^207^Pb*/^206^Pb* dates. Solid symbols near the *x*-axis represent the actual uncertainties of the ^238^U/^235^U ratios reported in the respective studies.

Measurements of ^238^U/^235^U ratios are burdened with their own uncertainty, which must be propagated into the total uncertainty of Pb–Pb ages. Since Pb and U isotopic measurements are independent, the age uncertainty components related to these measurements can be added in quadrature. The relationship between these uncertainty components in Pb–Pb meteorite chronology is shown in Fig. 2. If the age uncertainty contributed by the U isotopic measurement is equal to the age uncertainty from Pb–Pb isochron regression (diagonal line in the figure), then the total uncertainty would be equal to the uncertainty from isochron regression multiplied by √2 (∼1.41). In order to achieve equal uncertainty contribution for today's state-of-the-art precision of Pb isotopic dates of 0.1–0.3 Ma (Fig. 1c), we need precision of ^238^U/^235^U ratios of ∼0.007%–0.021%. The examples presented in Fig. 2 show that the uncertainties of U isotope measurements in some modern studies constitute a significant, and even dominant, part of the total age uncertainty, with [25] as an example where U isotope uncertainty is far less than Pb isotope age uncertainty. This problem is particularly prominent in the studies of chondrules and CAIs—small objects with low U content that just do not contain enough U for sufficiently precise ^238^U/^235^U measurements.

An alternative to individual U isotope analysis of meteorites and their components is assuming that the ^238^U/^235^U ratios are uniform in a certain class of meteorites, and using average values (with their uncertainties) in Pb–Pb age calculations. This approach was adopted for most chondrules in the studies of chondrules [11,26], and achondrites with exceptionally low U concentrations, e.g. [27]. The downside of this approach is that the assumption of uniform U isotopic composition is hard to verify.

More selective ^238^U/^235^U measurements require greater sensitivity to enable analysis of smaller quantities of U without prohibitive loss of precision, e.g. [28]. This can be achieved by increasing total ion yield (mainly restricted by transmission) beyond today's best values of ∼3%–4%, and by reducing detector noise without compromising fast response of the electrometers, to make the measurements independent of the ion beam fluctuations (see the discussion in [24]).

### Standardisation and inter-laboratory comparisons in Pb isotopic studies

Consistency of isotopic dates obtained using a variety of instruments, spikes and analytical protocols can be verified by analysis of reference materials (RMs) that emulate extraterrestrial materials. Standardisation in Pb–isotope extraterrestrial chronology has been historically performed using NIST SRM 981 and SRM 982 [29]. These RMs have been widely used by geochemistry, cosmochemistry and geochronology communities. However, these RMs do not represent ancient radiogenic Pb and cannot be used for age calculations.

To overcome the limitations of existing RMs, a series of synthetic solutions, dubbed EarlyTime solutions (note it is different from EarthTime solutions used by the zircon geochronology community), have been prepared that define a linear array in ^207^Pb/^206^Pb vs. ^204^Pb/^206^Pb space [30]. Analysis of these solutions allows construction of a simulated ‘isochron’ approximately corresponding to the age of the solar system, similarly to analysis of natural samples containing ancient radiogenic Pb and initial Pb. The use of the EarlyTime RMs has been gradually taking hold [13,25,31] and should be further expanded.

### Decontamination in Pb isotopic dating and its side effects

Reliable ages can be calculated either by direct measurement of the radiogenic ^207^Pb*/^206^Pb* ratio, or by construction of a ^207^Pb/^206^Pb vs. ^204^Pb/^206^Pb isochron. Step leaching in acids removes multiple components of non-radiogenic Pb that interfere with isochron construction. It has been found, however, that radiogenic Pb that is partially extracted from the minerals in some achondrites and CAIs has fractionated ^207^Pb*/^206^Pb* ratio [32]. If leaching-induced isotope fractionation of radiogenic ^207^Pb* and ^206^Pb* is unchecked, it can produce age biases up to 1–2 Ma. There are currently no viable alternatives to using acid leaching for removal of non-radiogenic Pb, but it is important to be aware of its possible side effects.

### Progress in MC-ICPMS and secondary ion mass spectrometry techniques

Development of more capable mass spectrometers fuelled analytical enhancements in other isotope chronometers employed in ESS studies. MC-ICPMS and secondary ion mass spectrometry (SIMS) are utilised for ^26^Al–^26^Mg, ^53^Mn–^53^Cr, ^182^Hf–^182^W, ^92^Nb–^92^Zr and other extinct radionuclide systems.

The most important innovation in MC-ICPMS has been the development of high-efficiency ion sources that increased the instrument transmission to up to ∼3%–4% (discussed in the ‘^238^U/^235^U variations and the accuracy of Pb isotopic ages’ section). The recently developed MC-ICPMS Sapphire from Nu Instruments, and Neoma from Thermo Fisher Scientific, are also equipped with more effective collision cells for elimination of molecular interferences with minimum loss of sensitivity. These instruments could be suitable for isotope analysis of Mg from single grains of Al-rich, Mg-poor minerals for precise ^26^Al–^26^Mg dating.

The progress of SIMS has included broader use of multicollector systems, and development of brighter primary ion sources, which enable analysis with higher secondary ion counting statistics, and hence higher precision of the dates. Modern SIMS-based ^26^Al–^26^Mg chronology of CAIs and achondrites can yield precision of the dates down to 0.05 Ma [33], and under favourable conditions, better than 0.02 Ma [34]. Another development that improved the accuracy of SIMS isotope analysis is in-depth study of the nature and extent of matrix effects in measuring elemental ratios, and synthesis of reference materials with a range of compositions more closely matching studied natural minerals to reduce matrix-related biases [35–38]. Discovering and eliminating data-processing artefacts that occur at low count rates [39] also improves SIMS data accuracy.

### The influence of isotope fractionation on age dating

Mass-dependent isotope fractionation is now known to be pervasive in all elements regardless of their atomic mass, e.g. [40]. The significance of ^238^U/^235^U variations for U–Pb age determination is now recognised by the geochronology and cosmochronology communities (‘^238^U/^235^U variations and the accuracy of Pb isotopic ages’ section), but the impact of stable isotope variations on age determinations with other chronometer systems is poorly understood. The general theoretical framework for age determination in the presence of natural isotopic fractionation has been presented by Di *et al.* [41]. The systematic uncertainty in the ages introduced by mass-dependent fractionation can be significant in some cases, but can be corrected with the proposed general formalism if the magnitude of natural fractionation is known.

## ISOTOPE CHRONOMETERS USED IN ESS STUDIES

### Which chronometers?

The solar protoplanetary disc contained numerous radionuclides, including 15 nuclides with half-lives between ∼100 ka and 100 Ma, listed in the sequence of increasing half-life: ^41^Ca, ^36^Cl, ^26^Al, ^10^Be, ^135^Cs, ^60^Fe, ^53^Mn, ^107^Pd, ^182^Hf, ^247^Cm, ^129^I, ^205^Pb, ^92^Nb, ^244^Pu and ^146^Sm (see Table S1, presented as Supplementary data online due to its large size). These nuclides have completely decayed (have become extinct) since the formation of the solar system, but variations in isotopic abundances of their decay products can be used for measuring time intervals of the nebular and early planetary evolution. The properties and usability of these chronometers also depend on abundance of parent nuclide and daughter nuclides, physical properties (e.g. volatility) and chemical and petrological affinities of the parent and daughter elements, and on our ability to precisely measure concentrations and isotopic ratios of these elements.

Three extinct radionuclide chronometers (^26^Al–^26^Mg, ^53^Mn–^53^Cr, ^182^Hf–^182^W) are established mainstream research tools in ESS studies, due to their versatility, relatively high abundance ratios of parent to daughter radionuclide abundances and hence substantial radiogenic effects in many types of extraterrestrial rocks and minerals, and the range of half-lives conveniently covering the range of time from the first solids to formation of planetary embryos and possibly planets. Recent reviews of the ESS chronology are entirely [6,42] or mainly [3,5] based on using these isotopic chronometers. Together with ^207^Pb*/^206^Pb, these systems have become the core group of methods in modern ESS chronology.

The other chronometers (^41^Ca–^41^K, ^10^Be–^10^B, ^36^Cl–^36^S, ^92^Nb–^92^Zr, ^107^Pd–^107^Ag, ^205^Pb–^205^Tl, ^129^I–^129^Xe, ^146^Sm–^142^Nd and the systems based on decay of ^244^Pu) can provide information about the processes that don't fractionate parent/daughter element ratios of the main chronometer group, or those that occurred outside of the optimum time range of the main group. These chronometers have been reviewed by Davis and McKeegan [3] and Davis [5]. Some of them can be used for studying very early nebular processes (^41^Ca–^41^K), or irradiation in the disc (^36^Cl–^36^S, ^10^Be–^10^B). The system based on decay of the longest-lived known extinct radionuclide (^146^Sm to ^142^Nd) has become an important instrument for studying planetary differentiation in the first billion years after solar system formation. The system based on decay of ^244^Pu plays an important role in deciphering the timescale for the delivery of volatiles to planetary objects and their subsequent migration between planetary reservoirs [43].

Chronometers based on decay of the extant radionuclides U–Th–Pb, ^87^Rb–^87^Sr, ^147^Sm–^143^Nd, ^40^Ar–^39^Ar, ^176^Lu–^176^Hf, ^187^Re–^187^Os and U–Th–He are widely used in terrestrial and other planetary studies. Their application to ESS studies has been reviewed by Wadhwa [4]. Their special value is the possibility of direct and seamless linking of early planetary timescales and the geological timescale. These systems (with the exception of ^207^Pb*/^206^Pb*) usually yield dates that are insufficiently precise for direct use in ESS chronology, but their application to asteroidal and early planetary rocks provides clues to the chemical evolution of their parent bodies, as well as information about possible late disturbances.

^87^Rb–^87^Sr is also used in the form of an initial Sr chronometer, where the ^87^Sr/^86^Sr ratio measured in a material with a very low Rb/Sr ratio (e.g. CAIs that experienced melting, some achondrites and their minerals, and chondritic phosphates) is projected back to the evolution line of their source reservoir with high Rb/Sr typical of the solar nebula and primitive chondrites. Application of the initial Sr chronometer to CAIs and achondrites yields the time estimate for the loss of volatile elements that accompanies separation of the condensing solids from the nebula, and thus sets the older limit on the time of accretion [44,45]. Initial ^87^Sr/^86^Sr in chondritic phosphates can be used to evaluate the time of thermal metamorphism of chondrites [46,47]. The time resolution of the initial ^87^Sr/^86^Sr chronometer depends on precision of Sr isotope analysis. Recent advances in precision of Sr isotope analysis, in particular refining the dynamic multicollector TIMS procedure [45,48,49] bring precision of the initial Sr chronometer into the typical precision range of ^53^Mn–^53^Cr, ^182^Hf–^182^W and Pb–Pb methods.

### Initial abundance of parent radionuclides: homogeneous or heterogeneous?

Calculation of an isotopic age requires knowledge of the abundance of the parent radionuclide, which is usually expressed with the atomic abundance ratio of that nuclide to a stable isotope of the same element (e.g. ^87^Rb/^85^Rb, ^26^Al/^27^Al) or, in the case of the U–Pb system, the abundance ratio of parent radionuclides of two decay schemes (i.e. ^238^U/^235^U). For long-lived chronometers, we can get this information by direct isotope analysis of the parent element. For short-lived chronometers, where the parent nuclide is completely extinct, this is not possible. If we construct ‘fossil isochrons’ such as ^26^Mg/^24^Mg vs. ^27^Al/^24^Mg, for two ESS materials using the same extinct radionuclide system, the time difference calculated from isochron slopes will be accurate only if both materials were formed from a reservoir where the abundance of the parent nuclide (^26^Al/^27^Al) changes only with time due to radioactive decay, but is uniform at any given moment.

Homogeneity of extinct radionuclide distribution can be tested by dating pairs of events with two chronometers: one that is being tested, and a reference one. The requirements for analytical quality of dating, and for geochemical integrity of the chronometer systems, are very high, and are similar to those in determination of half-lives by geological age comparison [50]; both isotopic clocks must be completely reset in the same, practically instantaneous, event, the parent and daughter nuclides must be completely retained ever since, and the radiogenic effects must be large enough to allow precise and unequivocal calculation of well-resolved time intervals. Few ESS materials satisfy these requirements, but there are some age determinations that come close enough to indicate that at least two extinct radionuclides have been distributed heterogeneously.

The evidence for heterogeneous ^26^Al/^27^Al distribution has been articulated by Krestianinov *et al.* [25]. There are four pairs of materials for which ^26^Al–^26^Mg and U-corrected ^207^Pb*/^206^Pb* age intervals are clearly mismatched: volcanic angrites vs. CAIs [51]; chondrules vs. CAIs [52]; volcanic angrites vs. Erg Chech 002 [25]; and Erg Chech 002 vs. CAIs [53]. Taken at face value, these data suggest that ESS materials can be broadly divided into three groups with initial ^26^Al/^27^Al at the time of the solar system formation being (i) around 5.23 × 10^−5^ (CAIs), (ii) around 1 × 10^−5^–2 × 10^−5^ (volcanic angrites and some chondrules) and (iii) intermediate values (another group of chondrules, Erg Chech 002, and possibly some other achondrites); see Fig. 2 in [25]. Interpretation of the age interval mismatches as the evidence for ^26^Al/^27^Al heterogeneity is not universally accepted, and alternative interpretations have been proposed, which invoke inaccuracy of ^207^Pb*/^206^Pb* ages for CAIs [54,55] and also for some achondrites [56].

There is also emerging evidence for heterogeneous distribution of ^92^Nb, based on the difference between the solar system initial ^92^Nb/^93^Nb of 1.66 × 10^−5^ (±0.10 × 10^−5^) determined by Haba *et al.* [57] by ^92^Nb–^92^Zr and U–Pb analyses of zircon and rutile from mesosiderites, and the value of 2.96 × 10^−5^ (±0.27 × 10^−5^) derived in [58] from an internal ^92^Nb–^92^Zr isochron for ungrouped achondrite NWA 6704, precisely dated with U–Pb, ^26^Al–^26^Mg and ^53^Mn–^53^Cr methods by Amelin *et al.* [59] and Sanborn *et al.* [60]. The difference in initial ^92^Nb/^93^Nb is explained by Hibiya *et al.* [58] as reflecting enrichment in ^92^Nb from a nearby supernova in the outer part of the solar protoplanetary disc, where the parent body of NWA 6704 probably formed. ^92^Nb–^92^Zr dating of ungrouped achondrite Erg Chech 002 [61] yielded the initial ^92^Nb/^93^Nb of 2.79 × 10^−5^ (±0.47 × 10^−5^), similar to the value for NWA 6704 determined by both Hibiya *et al.* [58] and Mane *et al.* [61]. These data reinforce the evidence for heterogeneous distribution of ^92^Nb, but also show that the pattern of this distribution does not necessarily correlate with the origin of the meteorite parent bodies in the inner vs. outer parts of the protoplanetary disc.

While the evidence for some heterogeneity of ^26^Al and ^92^Nb appears reasonably well justified, the pattern and overall extent of heterogeneity remain to be determined. It is currently unclear how much ^26^Al and ^92^Nb extinct radionuclide chronometry is affected by this heterogeneity. If it is firmly established that the distribution within a certain class of meteoritic and/or planetary materials is homogeneous, then age comparisons among any materials in this class can be performed with the usual dating formalism that assumes homogeneous distribution. For age comparison between the groups of materials with different initial abundances we have to account for these differences. There is currently no direct evidence for heterogeneous distribution of the other extinct radionuclides, including the mainstream chronometers ^53^Mn [60] and ^182^Hf [62], so their use in ESS age dating can proceed with the usual assumption of homogeneous distribution and the best available estimates of the initial abundance.

### Half-lives of radionuclides

Accurate isotope chronology critically depends on precisely knowing accurate values of half-lives of parent radionuclides, because systematic uncertainties in the half-lives are directly propagated into systematic uncertainties of the ages. An evaluation of the status of half-lives of long-lived radioisotopes used in geochronology has been presented by Begemann *et al.* [50], and detailed assessment of the following progress in half-life determinations for ^87^Rb, uranium isotopes ^234^U, ^235^U and ^238^U, and Sm isotopes ^146^Sm and ^147^Sm is given in the reports of the IUPAC-IUGS joint Task Group ‘Isotopes in Geosciences’ [63–65]. A general overview of half-lives in the range between 30 and 10^8^ years, which includes all nuclides used in extinct chronometers, has been presented by Heinitz *et al.* [66].

New determinations of half-lives published in the last 20 years are summarised in Table 1. These data are obtained with a combination of advanced decay counting, careful control of radiochemical purity, and accurate concentration determination with isotope dilution mass spectrometry. We are not discussing the data for the isotopes assessed [63–65], with the exception of ^146^Sm, the situation for which changed significantly since publication of the report by Villa *et al.* [65].

**Table 1.** Recently revised half-lives (My) of extinct chronometer radionuclides.

Radio nuclide	Old value	±	Reference	New value	±	Reference	% change	Decrease of relative uncertainty
^60^Fe	1.49	0.27	[67]	2.62	0.04	[160]	76	11.87
^60^Fe	1.49	0.27	[67]	2.50	0.12	[161]	68	3.78
^60^Fe	1.49	0.27	[67]	2.69	0.28	[162]	81	1.74
^182^Hf	9	2	[163]	8.90	0.09	[164]	1.1	21.98
^41^Ca	0.103	0.007	[165,166]	0.0994	0.0015	[167]	3.2	4.60
^146^Sm	103	4.5	[168]	68	7	[75]	34	0.42
^146^Sm	103	4.5	[168]	92.0	2.6	[77]	11	1.54

For some nuclides shown in Table 1 (^182^Hf, ^41^Ca), new measurements confirm previously used values, but greatly reduce the uncertainty. The absolute values of the time intervals calculated with new half-life values for these systems remain unchanged within uncertainties, but more precisely known half-lives allow more accurate comparison of the dates determined with different decay systems. For the other nuclides (^60^Fe, ^146^Sm), the half-life values are changed well outside the combined uncertainties of both old and new determinations, and the time intervals calculated with these chronometers have drastically changed as a result.

For the ^60^Fe–^60^Ni system, new determinations of half-life and initial abundance of ^60^Fe changed the status of this system in cosmochemistry. The half-life of 1.49 Ma [67] and initial abundance estimates for ^60^Fe that were available 20–30 years ago suggested [68,69] that this radionuclide was a major heat source in the ESS (second in the amount of heat production after ^26^Al), and is a capable chronometer system. However, the new longer half-life of ∼2.6 Ma, confirmed in three independent studies (Table 1), together with the downwards revision of the initial ^60^Fe/^56^Fe ratio [70–72] suggest that the importance of ^60^Fe as a chronometer and as an ESS heat source are not as significant as previously thought.

The half-life value of 103 Ma, based on consistent determinations reported by Nurmia *et al.* [73] and Friedman *et al.* [74] has been used by cosmochemists and researchers of the early earth for ^146^Sm decay since the early days of the ^146^Sm–^142^Nd system (see [65] for details). Publication of the half-life value of 68 ± 7 Ma in 2012 [75] introduced an ∼30% uncertainty of the decay rate of ^146^Sm and created a major disturbance in using this decay scheme. Villa *et al.* [65] did not find any of the published values convincing enough to serve as a basis for the IUPAC-IUGS Task Group recommendation, and suggested that the researchers using the ^146^Sm–^142^Nd system should perform a twin set of calculations with both half-lives. In 2023, the authors of [75] retracted it [76], quoting a likely flaw in the chemical processing of the Sm samples as a reason for retraction. A recently published paper [77] reported a new ^146^Sm half-life determination that yielded 92.0 Ma ± 2.6 Ma. Using this value introduces an ∼11% change in calculated time intervals compared to the previously adopted value (Table 1). Progress reports for another ongoing ^146^Sm half-life determination have also been published [78,79], but no half-life value from that study has been released yet.

## MORE AND BETTER ROCKS TO STUDY

Two types of extraterrestrial rocks are available for research, including age dating: (i) samples delivered by space missions from planetary bodies, asteroids and comets; and (ii) meteorites—the remains of the rock fragments that were excavated by impact from their parent bodies (planets or asteroids), sent in to Earth-crossing orbits by gravitational influence of other planets such as Jupiter, ablated during passage through the Earth's atmosphere, and landed on the Earth's surface.

Samples returned by space missions and meteorites have distinct benefits and limitations as probes for the ESS, and complement each other (Table 2). An important advantage of sample-return space missions is that the source body and the sampling locality are chosen during the design phase of the mission as necessary and as technologically feasible. Another advantage is that terrestrial contamination can be minimised by technical means and improved sample handling. The greatest limitation is that each mission is a major undertaking requiring major allocation of scientific, engineering, financial and political resources. Because of this resource-intensive nature of space missions, only a small number of bodies can be sampled and subsequently studied.

**Table 2.** **Strengths** and weaknesses of meteorites and sample-return space missions as space probes.

	Meteorites	Sample-return missions
Source body and sampling location	Guesstimate	**Known**
Number of parent bodies sampled	**Many**	Very few
Amount of material	Small to large	Small to large
Cost	**Low to moderate**	High to very high
Terrestrial processing	Low to high	**Low to negligible, and controlled**

The main benefit of meteorites as space probes is their exceptional diversity. Over 70 000 meteorites available in collections probably come from about 100 or more parent bodies (Table 3). This diversity is unlikely to be matched by space missions in the foreseeable future. The cost of meteorites is many orders of magnitude lower than the cost of rocks from sample-return missions, freeing up resources that can be invested into analytical instrumentation and facilities. A limitation of meteorites as space probes is that their source bodies are unknown and can be only ‘guesstimated’ on the basis of indirect evidence. The rate of terrestrial alteration in meteorites varies broadly from nearly pristine (e.g. meteorites collected immediately after observed falls) to severe, caused by prolonged contact with terrestrial soils, groundwater and living organisms.

**Table 3.** Numbers of meteorites and their parent bodies.

	Number	Comments and references
Meteorites—total	45 458	6 April 2013 (Meteoritical Bulletin)
Meteorites—total	53 676	9 January 2017 (Meteoritical Bulletin)
Meteorites—total	67 442	14 January 2021 (Meteoritical Bulletin)
Meteorites—total	73 436	16 January 2024 (Meteoritical Bulletin)
Meteorites—total	76 909	21 December 2024 (Meteoritical Bulletin)
Parent bodies (PBs)—total	95–148	[169]
PBs of chondrites	∼15	[170]
PBs of achondrites	∼35	ibid
PBs of iron meteorites	∼60	ibid

Returned samples and meteorites are also similar in some ways. Both have experienced pre-terrestrial history of secondary processes involving impacts and exposure to cosmic rays. The amount of material can vary widely, from less than a milligram to tens and even hundreds of kilograms.

The last 20 years have been marked by spectacular progress in sample-returning space missions. The mission Hayabusa brought back ∼60 mg of rock from asteroid 25143 Itokawa in 2010, Hayabusa 2 brought back ∼5.4 g from asteroid 162173 Ryugu in 2020, and Osiris Rex brought back ∼249 g from asteroid 101955 Bennu in 2023. Due to the small size of the Itokawa sample, the opportunities for isotopic dating were limited, but petrological, geochemical and oxygen isotope data [80,81] clearly demonstrated close resemblance to thermally metamorphosed LL-type ordinary chondrites, thereby providing asteroidal context for chondrite chronology. For an almost 100 times larger sample from Ryugu, it was possible to set up a comprehensive programme of geochemical characterisation, including detailed chronological study. The nature of these rocks, similar to CI-type carbonaceous chondrites (CCs), is unfavourable for chronological studies due to scarcity of suitable minerals, but it was possible to determine the timing of aqueous activity on the asteroid Ryugu. ^53^Mn–^53^Cr isochron for dolomite measured using SIMS with the slope of 3.14 × 10^−6^ (±0.28 × 10^−6^) yielded the age of dolomite precipitation of 5.2 (+0.8/−0.7) Ma after the CAI formation [82], which is similar to the time of extensive aqueous alteration at 5.2 (+1.8/−1.4) Ma derived from analysis of multiple rock fragments [83]. McCain *et al.* [37] obtained steeper ^53^Mn–^53^Cr isochrons for dolomite and magnetite in a SIMS study of two Ryugu specimens, corresponding to 6.1 × 10^−6^ (±0.9 × 10^−6^) and 6.8 × 10^−6^ (±0.5 × 10^−6^). These isochrons would indicate that fluid–rock interaction in the Ryugu precursor planetesimal occurred within the first 1.8 million years of solar system history. The studies of Yokoyama *et al.* [82,83] and McCain *et al.* [37] use different approaches to the account for matrix-dependent relative sensitivity factors. Resolution of this age discrepancy requires ^53^Mn–^53^Cr dating of Ryugu secondary minerals using a more robust technique in which matrix effects are minimal or absent.

For Bennu, it would be premature to review chronological findings but, considering the CI-chondrite affinity of these samples, we can expect that the petrological restrictions on dating of Ryugu samples will also apply to Bennu. On the other hand, an almost 50 times greater sample size provides more room for finding low-abundance components, both primary and secondary, that would be more suitable for isotopic dating.

The pool of meteorites available for research has also grown significantly in recent years (see the change of the numbers of meteorites registered in the Meteoritical Bulletin Database over time in Table 3). Meteorites have been the main source of information on the ESS processes since the beginning of modern cosmochemistry. In the early days, chronological studies were mainly using the most common and easily available meteorites, such as eucrites and equilibrated ordinary chondrites. Eventually it became clear that the geological histories of these samples were too long and too complex to allow precise linking of isotopic dates to the stages of ESS evolution.

Modern ESS chronology is based on the studies of three groups of materials: (i) a relatively small number of exceptionally old and well-preserved meteorites such as angrites, eucrite-like meteorites that do not originate from asteroid Vesta, and some unclassified basaltic achondrites; (ii) chondrules from well-preserved unequilibrated ordinary and CCs; and (iii) CAIs and amoeboid olivine aggregates (AOAs). The current status of chronological studies of these materials is reviewed below in the ‘Ages of solids in the ESS—from dust particles to planets’ section.

**Figure 3.**
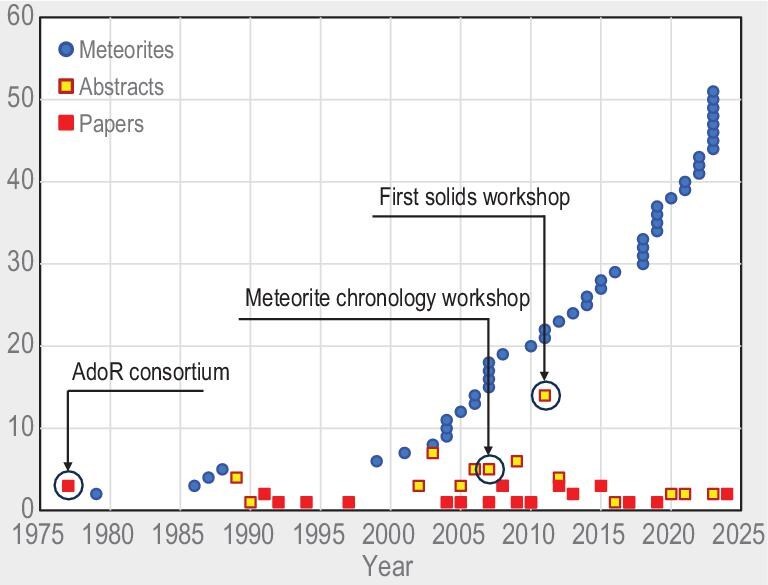
Total number of known angrites (blue), and number of published peer-reviewed papers (red) and abstracts (yellow) on angrite chronology per year.

With the recent increase of meteorite numbers, some of the previously small groups are now populated with numerous meteorites with diverse properties, allowing a detailed study of the history of their parent bodies. This can be illustrated with the data for angrites—a group of differentiated achondrites that attract steady interest as keepers of the record of earliest magmatism and volatile depletion, being also relatively pristine and amenable to precise isotopic dating with many chronometer systems. The total number of known angrites (based on the data from the Meteoritical Bulletin), and the number of published papers and abstracts on angrite chronology per year are shown in Fig. 3. The number of available angrites has grown significantly, and petrological studies [84] (and references therein) show that structural and compositional diversity of angrites is much greater than it appeared 10–15 years ago [85]. Unfortunately, most newly found angrites, including those that significantly differ from previously known samples, have not been dated yet. Chronological studies of angrites are reviewed in the ‘Ages of solids in the ESS—from dust particles to planets’ section.

## WHICH PROCESSES ARE WE DATING? LINKING PETROLOGY AND ISOTOPIC AGES

Isotopic chronometers measure the timing of fractionation between their parent and daughter radionuclides. Only the processes that fractionate these elements can be directly dated. The datable processes include condensation (volatility-induced fractionation), melt crystallisation (fractionation driven by crystal-melt partitioning), metasomatism (fractionation driven by solubility in fluids) and silicate–metal separation. Accretion and planetesimal collisions can be dated only indirectly. Metamorphism can be dated directly if there is a new mineral growth, or complete resetting of isotopic clocks. If the duration of metamorphic processes is long compared to the precision of dating, the interpretation of the dates relies on models of cooling and isotopic closure. In comparing isotopic dates of meteorites to each other, it is important to recognise the processes behind these dates, and to remember that different isotopic systems and different scales of sampling (e.g. whole rock vs. mineral grain vs. ion microprobe or laser ablation spot) can date different processes within the same meteorite. Without identifying these processes, ‘ages’ of meteorites with complex histories can be meaningless.

The processes that are being dated depend on the chemical properties and mineral affinity of the parent and daughter elements, and on the chemical and mineral composition of the rock (Table 4). If parent–daughter fractionation occurred in one process, interpretation of the ages is reasonably straightforward. The problems arise if several events contributed to the parent–daughter fractionation.

**Table 4.** The processes we date with the isotopic geo- and cosmo-chronometers.

Chronometer	Rock or component	Minerals	Process being dated
^235^U/^238^U– ^207^Pb/^206^Pb	Planetary igneous rocks	Zircon and baddeleyite	Melt crystallisation
	Planetary sedimentary rocks	Detrital zircon, monazite and other minerals	Crystallisation of source rocks
	Igneous meteorites and components (CAIs, chondrules, achondrites)	CAIs: all minerals Chondrules: Ca-rich pyroxene and mesostasis Achondrites: all minerals	Beginning of Pb retention upon cooling If cooling is fast, approximates the time of melt crystallisation; if cooling is slow, yields closure of Pb diffusion in silicate minerals (usually > 900 K)
	Metamorphic minerals in planetary rocks and meteorites	Apatite, silicic apatite, merrillite, titanite, rutile, garnet	Closure of Pb diffusion (usually <900 K in phosphate minerals)
^87^Rb–^87^Sr	Planetary felsic (meta)-igneous rocks—whole rock dating	Bulk rocks	Igneous differentiation during protolith formation (imperfect chronometer, superseded by zircon U–Pb)
	Planetary felsic igneous rocks—minerals	Mica (muscovite, biotite, phlogopite), K-feldspar	Closure to diffusion (approximates the time of igneous crystallisation if cooling was fast)
	Chondrules	Bulk chondrules	Primitive chondrites—chondrule formation (melting of nebular condensates); equilibrated (metamorphosed) chondrites—metamorphism
^40^K–^40^Ar	Planetary igneous rocks	Bulk rock and K-bearing minerals	Fast cooling—approximates crystallisation, slow cooling—closure for Ar diffusion
	Planetary metamorphic rocks	Bulk rock and K-bearing minerals	Closure for Ar diffusion after metamorphism
	Meteorites	Bulk rock and K-bearing minerals	Closure for Ar diffusion, or impact
^147^Sm–^143^Nd	Planetary felsic (meta)-igneous rocks—whole rock dating	Bulk rocks	Igneous differentiation during protolith formation (imperfect chronometer, superseded by zircon U–Pb)
	Planetary igneous rocks and meteorites—minerals	Olivine, pyroxenes, feldspar, phosphates	Igneous crystallisation if cooling was fast, closure for diffusion if cooling was slow
	Planetary metamorphic rocks	Garnet	Growth of garnet crystals or certain domains within crystals, or closure for diffusion
^26^Al–^26^Mg	Bulk CAIs, chondrules and primitive (undifferentiated) achondrites	Bulk materials	Al/Mg fractionation in the solar nebula, or partial evaporation of Mg during re-heating
	Bulk differentiated igneous meteorites	Bulk rocks	Differentiation of the parent body (approximates the time of accretion), or crystal fractionation in the magma chamber (approximates the time of magmatism)
	Minerals in CAIs, chondrules and igneous meteorites	Hibonite, spinel, pyroxene, melilite, anorthite	Crystallisation of the melt, or re-crystallisation; may be reset by solid-state diffusion
^53^Mn–^53^Cr	Bulk chondrules and primitive (undifferentiated) achondrites	Bulk materials	Mn/Cr fractionation in the solar nebula
	Bulk differentiated igneous meteorites	Bulk rocks	Differentiation of the parent body, or crystal fractionation in the magma chamber
	Minerals in chondrules and igneous meteorites	Olivine, kirschsteinite, pyroxene, chromite, spinel, ilmenite	Crystallisation of the melt, or re-crystallisation; may be reset by solid-state diffusion
	Secondary minerals in CCs	Fayalite, carbonates	Aqueous alteration in the parent body
^182^Hf–^182^W	CAIs	Bulk CAIs	Hf/W fractionation in the solar nebula
	Chondrites and their fractions	Bulk chondrules or chondrule pools; silicate and metal fractions	Metal–silicate segregation, approximates accretion
	Bulk differentiated igneous meteorites	Bulk rocks	Metal–silicate segregation in the parent body
	Minerals in igneous meteorites and CAIs	Pyroxene, olivine, bulk silicate fractions, metal	Crystallisation of the melt, or metamorphism
	Planetary rocks	Bulk rocks	Core formation, mantle differentiation (interpretation is model-dependent)
^146^Sm–^142^Nd	Planetary felsic (meta)-igneous rocks	Bulk rocks	Mantle differentiation (interpretation is model-dependent)
	Meteorites and the earliest planetary rocks—minerals	Olivine, pyroxenes, feldspar, phosphates	Igneous crystallisation if cooling was fast, closure for diffusion if cooling was slow
Initial ^87^Sr/^86^Sr	CAIs and achondrites	Bulk materials, plagioclase, phosphates	Separation of volatile-depleted solids from the nebula; approximates the beginning of accretion, strongly model-dependent
	Chondrites	Phosphates	Growth of phosphate crystals during metamorphism

**Figure 4.**
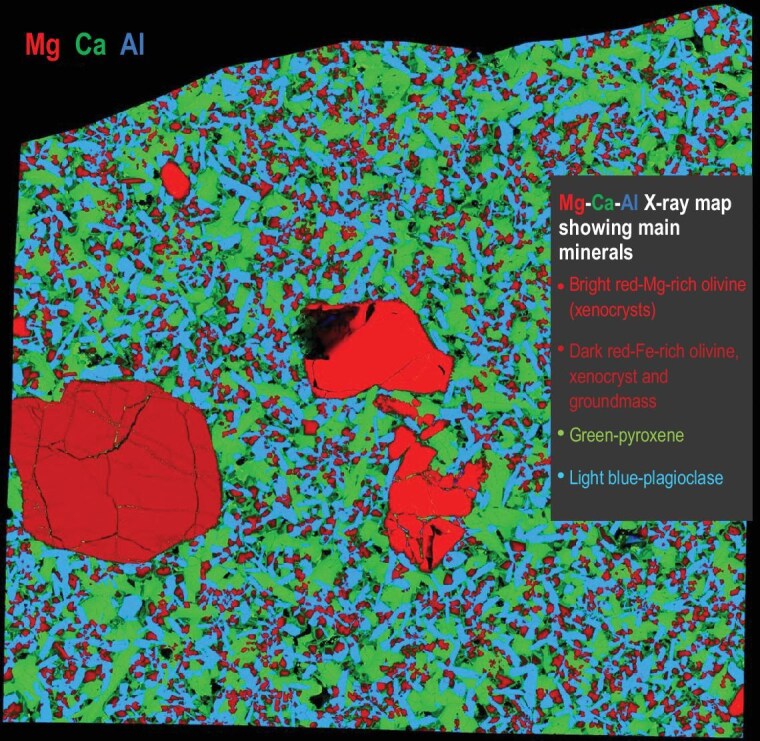
Olivine xenocrysts in rapidly cooled angrite Oued Namous 001 (from presentation by Amelin *et al.* at the Lunar and Planetary Science Conference in 2024 [171]).

Secondary processes, including thermal metamorphism, aqueous or hydrothermal alteration, impacts and exposure to terrestrial environments can change the ratios of parent to daughter isotopes. Interpretation of the ages is also complicated for igneous rocks that contain xenocrysts (minerals formed by earlier episodes of melting) or incompletely melted condensates. This complexity can affect all types of igneous objects that formed in the ESS: refractory inclusions (CAIs and AOAs), chondrules and achondrites. Recent data show that occurrence of xenocrysts in achondrites is common (e.g. olivine in volcanic angrites, Fig 4, and pyroxene in ungrouped achondrite Erg Chech 002 [34,86]). Unlike the role of secondary processes, the presence of xenocrysts is not yet widely recognised in the cosmochemistry community as a complicating factor in age determination. A recent study of the ^26^Al–^26^Mg system in spinel and plagioclase in volcanic angrite D'Orbigny [87] shows that it can be difficult to distinguish between the effects of secondary reworking and the presence of xenocrysts. Developing the strategy of reliable dating when either or both of these complicating factors can be present would require coordinated petrological and isotopic studies of a variety of meteorites.

## NUCLEOSYNTHETIC DICHOTOMY AND ESS CHRONOLOGY

One of the most important recent discoveries in cosmochemistry was finding a systematic difference in nucleosynthetic isotope signatures among meteorites, first recognised as a dichotomy in Ba, Nd, Sm, Cr, Ti and O isotope compositions between CCs and non-carbonaceous chondrites (NCs, e.g. ordinary and enstatite) [88–91]. Subsequent studies showed that this distinction is seen not only in chondrites, but also in achondrites, irons and stony-irons, and can observed in abundances of many isotopes, including ^30^Si, ^48^Ca, ^54^Fe, ^84^Sr, isotopes of Zr, Mo, W and other elements, e.g. [60,92–99]. Similar isotopic variability is also observed among meteorite components, e.g. chondrules, which shows nebula-wide mixing between CC and NC reservoirs [100]. Furthermore, there is significant heterogeneity within both CC and NC groups, seen in the studies cited above, and appearing increasingly complex as the amount and precision of the data increase, e.g. [101]. The origin of the CC–NC dichotomy has been attributed to different accretion location in the disc: the NC group materials originated in the inner disc, while the CC materials derived from the outer disc, and the NC and CC regions of the disc may have been separated due to early formation of proto-Jupiter [92], although consideration of both isotopic signatures and the content of volatile elements [102] shows that this relationship is not straightforward.

This nucleosynthetic variability implies that there were different domains in the ESS that have never been well mixed. In the currently available data, there are hints that the processes in the CC and NC domains occurred at different times. ^26^Al–^26^Mg chondrule dates in best-preserved chondrites (i.e. corresponding to the time of chondrule formation) show that chondrules in CCs appear to be generally younger than in ordinary chondrites [103–105], although there is an overlap between the age distributions. Accretion ages derived from thermal modelling [106] are younger for CCs than for NCs and achondrites, although these ages should be used with caution because the thermal model employs the assumption of homogeneous distribution of ^26^Al at the ‘canonical’ level (i.e. similar to the ^26^Al abundance in the main population of CV chondrite CAIs at the time of their formation). There is also difference in the time of crystallisation between the oldest achondrites with NC and CC nucleosynthetic affinities, discussed below in the ‘First 7 million years of the solar system as they appear in 2025’ section.

## THE PROSPECT OF ANCHOR-FREE MULTI-SYSTEM CHRONOLOGY

Integration of timescales based on decay of different extinct radionuclides usually employs ‘anchors’—materials that have been dated with many isotope chronometers, and are used as age references on the basis of an assumption that the readings of all chronometers correspond to the same event (e.g. melt crystallisation). ‘Anchors’ are currently widely used for linking timescales of two or more isotope chronometer systems (see, for example, the discussion of Krestianinov *et al.* [25]), but their use is only justified among the materials with homogeneous distribution of short-lived chronometer nuclides. If the solar protoplanetary disc contained several domains with internally homogeneous short-lived radionuclide distribution, then each domain requires its own anchor, and construction of a disc-wide timescale can be achieved by linking ‘local’ timescales using the ^238^U/^235^U-corrected ^207^Pb*/^206^Pb* ages of the anchors. Furthermore, the requirements for geochemical integrity of the ‘anchors’ are very high and are similar to the requirements for meteorites that are used for testing homogeneity of short-lived radionuclide distribution (‘Initial abundance of parent radionuclides: homogeneous or heterogeneous’ section above). Volcanic angrite D'Orbigny is the mostly commonly used ‘anchor’ material for ^26^Al–^26^Mg, ^53^Mn–^53^Cr, ^182^Hf–^182^W and U–Pb systems; however, its suitability has been questioned based on new petrological and isotopic data [87,107–109].

**Figure 5.**
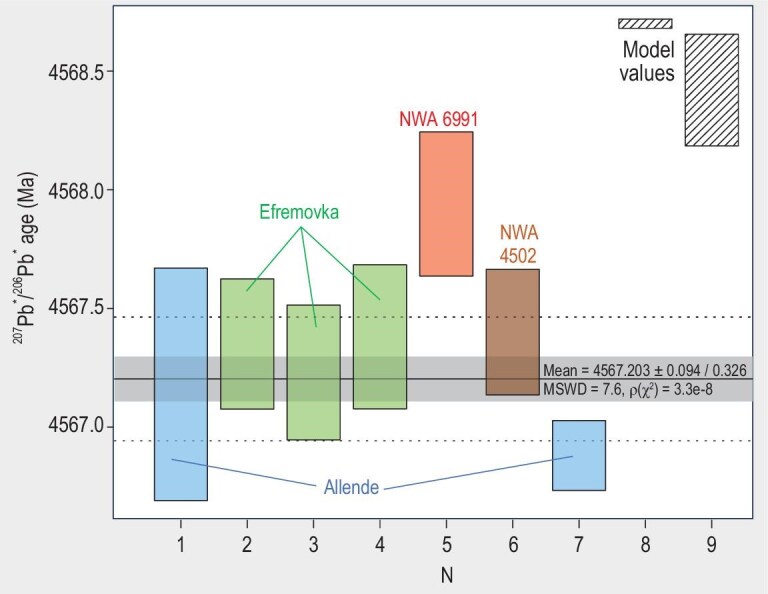
Published ^207^Pb*/^206^Pb* U-corrected ages of CAIs from CV chondrites. (1) Allende CAI SJ101 [20]; (2–4) Efremovka CAIs 22E, 31E and 32E [11]; (5) NWA 6991 CAI B4 [121,122]; (6) Combined isochron for NWA 4502 CAIs 1, 5, 6 and 7 [119,120]; (7) Weighted average of isochron ages for Allende CAIs CG-6, CG-10, CG-11, TS45 and SJ101 [123]; (8) Model-derived value from [55], no uncertainty assigned; (9) Model-derived value from [54]. All ages are shown exactly as presented in the respective publications.

There is a number of systematic discrepancies between age intervals of well-preserved ESS objects determined with ^207^Pb*/^206^Pb* and ^26^Al–^26^Mg chronometers, which complicate building a consistent timescale of solar system formation. Our preferred interpretation, discussed above in the ‘Initial abundance of parent radionuclides: homogeneous or heterogeneous’ section, is that these discrepancies are caused, at least in part, by heterogeneous distribution of ^26^Al. Alternative ‘anchor-free’ approaches to construction of unified chronometry of meteorites by compiling literature Al–Mg, Mn–Cr, Hf–W and Pb–Pb data for achondrites and applying statistical techniques to minimise the discrepancies between their times of formation across these systems [54,55,110] have been proposed. These approaches involve assumption of homogeneous distribution of extinct chronometer radionuclides. These attempts achieved their goal, but at a price; the currently accepted age of CAIs of 4567.30 ± 0.16 Ma [11] based on precise and consistent ^238^U/^235^U-corrected Pb–Pb isochrons for four CAIs (three from Efremovka and one from Allende CV chondrites) has to be dismissed in favour of the older CAI ages (Fig. 5) that are calculated as one of the model-derived free parameters, but have no direct verification. Future tests of both anchor-based and anchor-free approaches to construction of a unified ESS timescale will require new, high-precision dates, with supporting detailed petrological and isotopic data, for CAIs and the best-preserved ancient achondrites. It is important to point out that the heterogeneity of ^26^Al as argued for by Krestianinov *et al.* [25] does not depend on any particular CAI age and would hold regardless of assuming an anchor-based or anchor-free approach to ESS chronology.

## AGES OF SOLIDS IN THE ESS—FROM DUST PARTICLES TO PLANETS

In this section we review the best available modern age data for early system materials, selected and evaluated following the quality criteria and considering the pitfalls discussed above.

### The solids that (possibly) pre-date the solar system

Presolar grains are interstellar mineral grains from various stellar sources that have never been extensively equilibrated with the predominant solar system material. They are recognised by hugely anomalous isotopic composition of practically all constituent elements that cannot be plausibly produced by any processes, including fractionation, radioactive decay and irradiation, within the solar system [111].

Direct dating of presolar grains with any of the chronometers discussed above (‘Which chronometers?’ section) is a formidable challenge, either because these grains don't contain enough atoms of radioactive elements to produce measurable radiogenic effects, or because the abundance of radionuclides in the environments where these grains formed is unknown. Still, it was possible to estimate the timespan between formation and incorporation into the accreting solar system for some presolar silicon carbide grains from the Murchison CM chondrite [112]. The ages, based on cosmogenic ^21^Ne produced by exposure to the galactic cosmic rays, range from 3.9 ± 1.6 Ma to ∼3 ± 2 Ga before the beginning of the solar system.

Besides presolar grains, some chondrites contain isotopically anomalous platy hibonite crystals (PLACs), CAIs with fractionation and unidentified nuclear effects (FUN CAIs) and corundum-bearing, grossite-rich, hibonite-rich inclusions in many groups of chondrites. These materials had initial low ^26^Al/^27^Al of <5 × 10^−6^, pointing to young age, but also possess large isotopic anomalies in many elements, suggesting their formation before broad-scale homogenisation in the solar nebula, and hence an exceptionally old age [113]. The ^26^Al dilemma of PLACs and FUN CAIs can be resolved if these objects formed before the material containing freshly synthesised ^26^Al was injected in the solar nebula. In this case, the ^26^Al–^26^Mg chronometer is unapplicable to these objects. FUN CAIs STP-1 [114] and CMS-1 [115] from the Allende CV chondrite have very low U concentration, estimated at 0.38–1.2 ppb, which is about 30–300 times lower than in ‘normal’ CAIs. The Pb isotope composition in the CAI CMS-1 is unradiogenic, with ^206^Pb/^204^Pb between 10.6 and 16.8, and defines an imprecise isochron corresponding to 4586 ± 79 Ma [115]. Application of the ^182^Hf–^182^W chronometer to the FUN CAI STP-1 yielded a more conclusive result: an internal isochron yields initial ^182^Hf/^180^Hf of 9.6 × 10^−5^ (±1.1 × 10^−5^), which is indistinguishable from the isochron for ‘normal’ CAIs, ^182^Hf/^180^Hf = 9.72 × 10^−5^(±0.44 × 10^−5^) [116], suggesting simultaneous formation of ‘normal’ and FUN CAIs. To the best of our knowledge, there are currently no direct age data for PLACs.

### First 7 million years of the solar system as they appear in 2025

In this section we summarise recent progress in Pb isotopic dating of the solar system's oldest solids: CAIs, chondrules and the oldest achondrites. For each of these materials, we discuss available data, outstanding problems and possible ways of solving them. Only ^238^U/^235^U-supported ^207^Pb*/^206^Pb* ages are considered here. A summary of ^26^Al–^26^Mg, ^53^Mn–^53^Cr and ^182^Hf–^182^W chronology can be found in work by Davis [5] and Anand and Mezger [6].

Here, we discuss only the objects and events older than the arbitrary age limit of 4560 Ma, a conservative estimate of the time by which the gas and dust in the protoplanetary disc had accreted or dissipated, and protoplanets formed (cf. Morbidelli *et al.* [117]).

#### Ca–Al-rich refractory inclusions

The ages of CAIs from CV chondrites are plotted in Fig. 5. This compilation includes ages published in abstracts supported with ^238^U/^235^U ratios measured in the same CAIs, but excludes the value for CAI B1 from CV chondrite NWA 2364 [118], because the method of proxy correction for U isotope composition using Th/U ratio used in that study remains unproven. The weighted mean value of 4567.30 ± 0.16 Ma of four Efremovka and Allende CAI ages [11,20] has been accepted as the best estimate of the time of CAI formation. Subsequent publications (currently available only as abstracts) show that the picture is more complex. The age of 4567.40 ± 0.27 Ma, derived from a combined isochron for four CAIs from NWA 4502 [119] and corrected for their mean ^238^U/^235^U of 137.808 ± 0.019 (uniform among these CAIs [120]), agrees with the value from Connelly *et al.* [11]. The same cannot be said about the ages of CAI B4 from NWA 6991 ([121,122]), which are distinctly older, and the weighted average of isochron ages for Allende CAIs CG-6, CG-10, CG-11, TS45 and SJ101 [123], which is younger than the value of Connelly *et al.* [11].

The reason for these age discrepancies is currently unclear. It can be related to secondary processes (metamorphism, metasomatism, impacts and/or terrestrial weathering) that affected some meteorites more than others. The differences in calculated ages may also reflect variations in the time of CAI formation, such as suggested by ^26^Al–^26^Mg systematics of fine-grained CAIs in CV chondrites [124]. Distinguishing between these possibilities (which are also not mutually exclusive) would require new CAI studies that combine U–Pb and ^238^U/^235^U measurements, detailed petrological and geochemical examination, and ^26^Al–^26^Mg, ^41^Ca–^41^K and ^182^Hf–^182^W dating. Studying the distribution of U and radiogenic Pb between the minerals would help to better constrain the meaning of the Pb isotopic ages of CAIs. Measuring concentrations and isotope compositions of the elements that are abundant in the matrix but depleted in CAIs would help to evaluate the degree of exchange during metamorphism and/or metasomatism. Finally, the isotope composition of uranium in various mineral phases in CAIs should be measured to make sure that the ^238^U/^235^U values used in age calculations are accurate for the mineral phases where Pb isotopic compositions were measured.

#### Chondrules

Chondrules—solidified droplets of melt of broadly mafic or ultramafic composition, usually hundreds of microns to a few millimetres in diameter—are among the most abundant (∼20%–80% by volume) components in all types of chondrites except CI [125,126]. Their formation is described by nebular models of melting dust aggregates of early condensates in the protoplanetary disc by local heating events, and planetary models where chondrules are interpreted as formed by collision of solid and/or partially molten planetesimals or larger bodies [127–129].

Chondrule formation chronology mainly relies on the ^26^Al–^26^Mg system, in particular analysis of feldspar and Al-rich mesostasis ([103–105]; see also reviews by Davis and McKeegan [3,5] and Anand and Mezger [6]). For chondrules from primitive chondrites with a low degree of metamorphism, these data could yield the time of chondrule formation. ^26^Al–^26^Mg isochrons for metamorphosed chondrules correspond either to the time of peak metamorphism or a point of Mg diffusion closure during post-metamorphic cooling (in the case of high-temperature metamorphism), or yield intermediate (usually meaningless) time reading between chondrule formation and metamorphism. It is important to note, however, that all ^26^Al–^26^Mg relative chronology of chondrules relies on the assumption of uniform ^26^Al distribution in the ESS.

**Figure 6.**
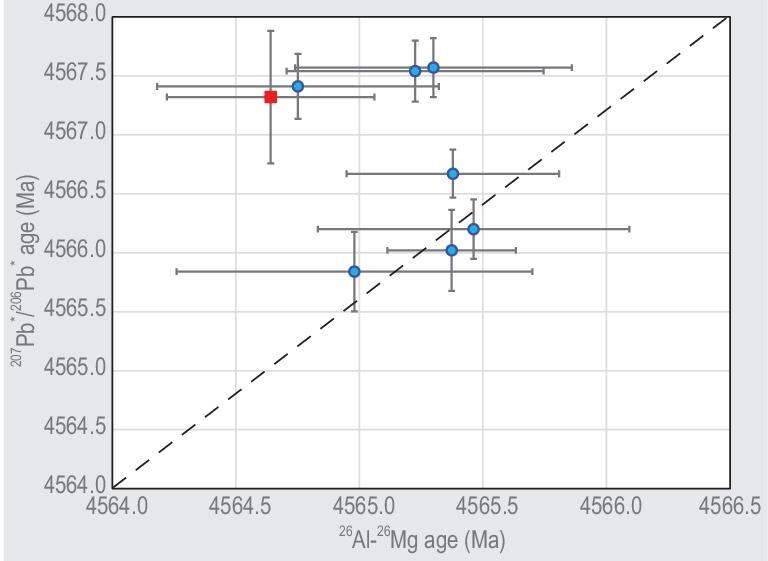
Ages of chondrules determined with ^26^Al–^26^Mg and ^207^Pb*/^206^Pb* methods, based on the data in Table 2 in [52]. Blue circles—chondrules from L3 chondrite NWA 5697; red square—a chondrule from CV chondrite Allende. ^26^Al–^26^Mg ages are calculated assuming the ‘canonical’ initial ^26^Al/^27^Al = 5.23 × 10^−5^ (±0.13 × 10^−5^) [172], the age of CAIs (i.e. the reference point of the solar system formation time scale) of 4567.3 ± 0.16 Ma [11] and uniform distribution of ^26^Al across CAI- and chondrule-forming regions. The dashed line shows the locus of equal ^26^Al–^26^Mg and ^207^Pb*/^206^Pb* ages.

Due to low U concentration (around average chondrite value of ∼10 ppb), only uncommonly large chondrules can be individually dated with the U–Pb method. Pb isotopic ages of 22 large chondrules from CV chondrite Allende, L3.1 chondrite NWA 5697 and CR2 chondrites NWA 6043 and NWA 7655 have been determined by constructing Pb–Pb isochrons using multi-step partial dissolution [11,26]. Six of these 22 chondrule ages are calculated with directly measured ^238^U/^235^U values, and the other 16 are calculated assuming average solar system ^238^U/^235^U of 137.786 ± 0.013 proposed by Connelly *et al.* [11]. Chondrules from NWA 5697 occupy the range of ages from 4567.61 ± 0.54 Ma to 4563.64 ± 0.51 Ma. Most of them (9 of 13) are older than 4566 Ma. The ages of chondrules from CR chondrites occupy a similar total range (from 4567.26 ± 0.37 Ma to 4563.24 ± 0.62 Ma). Taken at face value, these data imply that the epoch of chondrule formation started contemporaneously with CAI formation, and continued for about 4 million years. Considering correlation between the isochron ages and the initial ^207^Pb/^206^Pb ratios derived from intercepts of the isochrons and the Pb isotopic evolution curves, Bollard *et al.* [26] interpreted the spread of ages as evidence for early chondrule formation within 1 Ma after CAI formation followed by chondrule recycling. Subsequent ^26^Al–^26^Mg internal isochron dating of eight of these chondrules [52] showed that four NWA 5697 chondrules have consistent Pb–Pb and ^26^Al–^26^Mg ages, whereas for the other four chondrules (three from NWA 5697 and one from Allende) ^26^Al–^26^Mg ages are distinctly younger than Pb–Pb ages (Fig. 6). All these chondrules have ^26^Al–^26^Mg ages with overlapping uncertainties between 4565.5 and 4564.6 Ma, or 1.8–2.7 Ma after CAI formation. This interval is similar to the range of ^26^Al–^26^Mg ages of chondrules from a more extensive dataset for L and LL-type ordinary chondrites, e.g. Pape *et al.* [103]. Bollard *et al.* [52] interpreted the apparent disagreement between Pb–Pb and ^26^Al–^26^Mg ages as the evidence for reduced initial abundance of ^26^Al in the chondrule-forming region of the inner solar system. Desch *et al.* [54] re-examined Pb isotopic data of Bollard *et al.* [52] for four chondrules that have been analysed for ^238^U/^235^U ratios and, while generally confirming the age values, interpreted them as evidence that the crystallisation ages of CAIs are actually older than their Pb isotopic age. The true reason for Pb–Pb vs. ^26^Al–^26^Mg chondrule age discrepancy remains unclear. We think that heterogeneity of ^26^Al distribution probably contributes to this discrepancy, but this does not rule out the other possible causes. ^53^Mn–^53^Cr dating of chondrules [130] can help to resolve this discrepancy.

Bencubbin-type (CB) CCs contain chondrules that are unusually large, and depleted in volatile elements, which have been interpreted as formed from a vapour–melt plume produced by a giant impact between planetary embryos after dissipation of most dust in the protoplanetary disc [127]. Pb isotopic dating of CB chondrules has shown their unusually young age, initially determined at 4562.7 ± 0.5 Ma for CB chondrite Gujba, and 4562.8 ± 0.9 Ma for CB chondrite Hammadah al Hamra 237 [127]. Subsequent Pb isotopic dating of additional Gujba chondrules allowed refinement of their ages to 4562.49 ± 0.21 [131] and 4562.64 ± 0.13 Ma [132].

#### The oldest achondrites

Achondrites are igneous rocks that were formed by crystallisation of magma produced by melting on asteroids. Some achondrites (called primitive) have chemical compositions similar to chondrites, and are interpreted as the products of partial melting without separation of magma from the residual minerals. Other achondrites (called differentiated) have chemical and petrological characteristics typical for rocks produced by magma differentiation.

**Figure 7.**
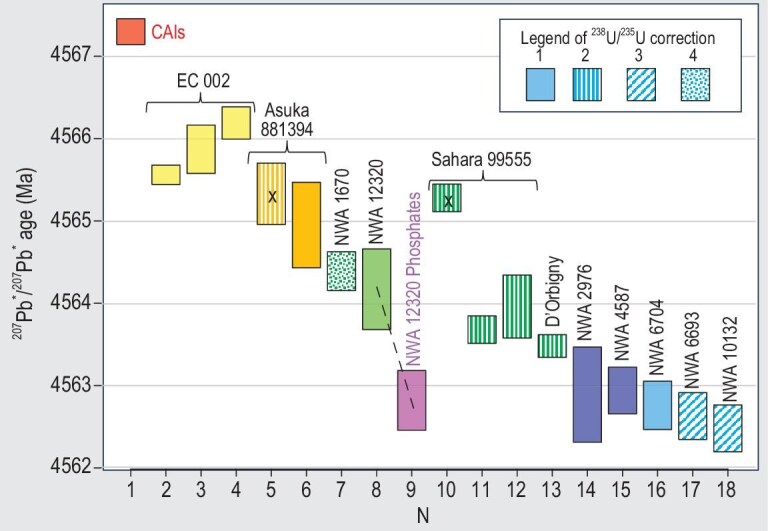
Published ^207^Pb*/^206^Pb* U-corrected ages of CAIs and achondrites. All ages are corrected for U isotopic composition as indicated in the embedded legend: (1, solid fill) ^238^U/^235^U measured in the same meteorite in the same study; (2, vertical stripes) ^238^U/^235^U measured in the same meteorite in another study; (3, diagonal stripes) ^238^U/^235^U measured in paired or closely related meteorite; (4, dotted fill) ^238^U/^235^U estimated from a broader group of meteorites. Colours: red—CAIs; yellow—Erg Chech 002; orange—Asuka 881394; green—rapidly cooled angrites; pink—phosphates in rapidly cooled angrite NWA 12320; dark blue—pairs of NWA 011; light blue—NWA 6704 and related meteorites. Sources of age data: (1) CAIs ([11]; (2) Erg Chech 002 [25]; (3) Erg Chech 002 [139]; (4) Erg Chech 002 [53]; (5) Asuka 881394 [136]; (6) Asuka 881394 [137]; (7) NWA 1670 [51]; (8) NWA 12320 silicate minerals [149]; (9) NWA 12320 phosphate minerals [149]; (10) Sahara 99555 [145]; (11) Sahara 99555 [9]; (12) Sahara 99555 [146]; (13) D'Orbigny [10]; (14) NWA 2976 [21]; (15) NWA 4587 [152]; (16) NWA 6704 [59]; (17) NWA 6693 [59]; (18) NWA 10132 [32]. Boxes marked with letter ‘x’ show the dates that have been superseded by later results.

Pb isotopic ages of the oldest known achondrites are summarised in Fig. 7. All the ages are corrected for ^238^U/^235^U variations. The ages published in peer-reviewed papers and abstracts are included. For clarity, the dates with uncertainties greater than ±1 Ma, preliminary results based on limited datasets, and the data for which correction for ^238^U/^235^U cannot be clearly established, are excluded.

In the last 25 years, two ungrouped achondrites—Asuka 881384 and Erg Chech 002 (EC 002)—have been considered candidates for the title of the oldest igneous rock in the solar system. Asuka 881394, a meteorite collected in Antarctica in 1988, was initially classified as a cumulate eucrite [133], but later was reclassified as an ungrouped achondrite due to a large difference in Δ^17^O from eucrites [134]. The exceptionally old age of Asuka 881394 was established by Nyquist *et al.* [135] on the basis of ^26^Al–^26^Mg and ^53^Mn–^53^Cr data, and was later confirmed by Pb isotopic dating [136]. The best current Pb isotopic age estimate for this meteorite is 4564.95 ± 0.53 Ma [137]. This value is based entirely on highly radiogenic data, and is therefore less affected by uncertainty in non-radiogenic Pb components than the Pb–Pb isochron age of Wadhwa *et al.* [136], which included some less radiogenic data in the regression.

EC 002, an ungrouped achondrite of andesitic composition, is currently considered the oldest known igneous rock in the solar system. This large (∼32 kg) meteorite, found in Algeria in 2020, is now one of the most extensively studied meteorites for petrology, geochemistry and isotope systematics ([86,138] and references therein), but its exact age remains elusive due to significant discrepancies among the published age data. The ancient ^26^Al–^26^Mg internal isochron age of EC 002 of 2.255 ± 0.013 Ma after CAIs (assuming that the two materials had identical initial ^26^Al/^27^Al) was discovered by Barrat *et al.* [34]. Three Pb isotopic ages [25,53,139], calculated using ^238^U/^235^U measured in each of these studies, vary from 4566.19 ± 0.20 to 4565.56 ± 0.12 Ma. Likewise, the slopes of ^26^Al–^26^Mg isochrons determined using SIMS [34,140] are about 30% lower than the slopes of the isochrons based on MC-ICPMS analyses [53,139,141]. This corresponds to the age difference of ∼0.45 Ma, about 20–30 times larger than the internal isochron age uncertainties. Yang *et al.* [142] obtained internal ^53^Mn–^53^Cr isochrons for two fragments of EC 002, which correspond to discrepant ages of 4567.3 ± 0.8 and 4565.9 ± 0.6 Ma. All these age discrepancies can be caused by a combination of secondary processes, the presence of xenocrysts described, e.g. by Barrat *et al.* [34] and Jin *et al.* [86], and possibly incompletely corrected systematic analytical uncertainties. Fortunately, future studies required for understanding age discrepancies of EC 002 are facilitated by the wide availability of this meteorite.

The oldest rapidly cooled angrites (sometimes called volcanic angrites, although impact melt origin of some of them is also possible), are ∼1–2 Ma younger than EC 002 and Asuka 881394. Antiquity of angrites has been known since the early chronological studies of Angra dos Reis (AdoR), the namesake of this meteorite group [143], and plutonic angrite Lewis Cliff 86010 [144]. An exceptionally old Pb–Pb age of 4566.18 ± 0.14 Ma (4565.28 Ma after correction for measured ^238^U/^235^U) was reported for rapidly cooled angrite Sahara 99555 by Baker *et al.* [145], but subsequent re-analysis at two different labs [9,146] showed that this meteorite is ∼1.3–1.6 Ma younger. The compositionally and mineralogically similar angrite D'Orbigny formed at 4563.48 ± 0.14 Ma (Pb isotopic age from [10], corrected using ^238^U/^235^U of Brennecka *et al.* and Tissot *et al.* [22,23] and Amelin *et al.* [32]), contemporaneously with Sahara 99555. D'Orbigny has been also precisely dated with ^53^Mn–^53^Cr [147], ^26^Al–^26^Mg ([51] and references therein) and ^182^Hf–^182^W [148], and is commonly used as an anchor sample for age comparison between extinct radionuclide chronometers. Suitability of D'Orbigny for this purpose has been recently questioned on the basis of the evidence for a disturbed ^147^Sm–^143^Nd system [107], suggested impact processing of some rapidly cooled angrites [108], and the possibility of chronological disturbance deduced from ^26^Al–^26^Mg systematics of spinel and plagioclase [87]. Rapidly cooled angrites NWA 1670 [51] and NWA 12320 [149] appear to be marginally older than D'Orbigny and Sahara 99555 (Fig. 7). Most rapidly cooled angrites found in the past 10 years have not been dated yet.

All achondrites discussed earlier in this section have NC nucleosynthetic affinity. Two small groups of ancient CC-type achondrites were also identified. The first are eucrite-like meteorites NWA 2976 and NWA 4587, paired with NWA 011 [150,151]. Pb isotopic dates of these meteorites, calculated with ^238^U/^23^ U ratios measured in the same studies, are 4562.89 ± 0.59 [21] and 4562.94 ± 0.29 Ma [152,153], respectively. The second group includes paired meteorites NWA 6693 and NWA 6704 [154,155]—unique achondrites with igneous cumulate mineralogy and chondritic abundance of the elements, and a closely related meteorite NWA 10132. Their Pb isotopic ages (calculated with U isotope composition measured in NWA 6704) are between 4262.76 ± 0.30 and 4262.48 ± 0.29 Ma [32,59]. The ages of all CC-type achondrites discussed here are indistinguishable from each other within uncertainty, and are ∼1–1.5 Ma younger than the ages of rapidly cooled angrites. Taken at face value, the combined age data for NC- and CC-type achondrites suggest that asteroidal magmatism in the outer solar system started ∼3 Ma later than in the inner solar system.

There are a number of ungrouped achondrites, for which existing chronological data suggest very early formation, e.g. NWA 7325, NWA 11119, Tafassasset and related meteorites. However, their previous chronology is either insufficiently precise because of very low concentration of U and radiogenic Pb, or is limited to extinct radionuclide systems and hence subject to uncertainties in chronometer cross-calibration.

The ages of the oldest eucrites are currently uncertain due to pervasive metamorphism. Recent dating of an unequilibrated eucrite NWA 8661 with exceptionally low secondary processing yielded a Pb isotopic age of 4563.44 ± 1.11 Ma [156], similar to the angrites D'Orbigny and Sahara 99555.

#### The time of planetesimal formation

Accretion of solids in a protoplanetary disc into larger aggregates and eventually into planetesimals, protoplanets and planets can be accompanied by fractionation of chemical elements, e.g. loss of volatiles due to heating, but it can also occur isochemically. In the latter case, the time of accretion can be bracketed between the times of two datable events. In the case of the parent bodies of chondrites, the older limit is set by the ages of the youngest chondrules, and the younger limit by the ages of the oldest secondary minerals that formed in the matrix by fluid circulation in the newly formed planetesimal. The latter approach can be used regardless of the presence of chondrules; for example, it was used in ^53^Mn–^53^Cr dating of secondary dolomite in the rocks from asteroid Ryugu [82,83]. It was found that dolomite precipitation occurred at 5.2 (+0.8/−0.7) Ma after CAI formation. This in turn means that the primary parent body of the rocks sampled by the Hayabusa 2 spacecraft on asteroid Ryugu had already accreted by that time. Formation of the primary parent body should not be confused with formation of the asteroid Ryugu, which is a rubble pile re-assembled from the fragments of earlier asteroids disrupted by collisions [157].

For asteroids that underwent melting, the older limit on the time of accretion can be estimated with the initial ^87^Sr/^86^Sr chronometer, which yields the time of loss of volatile elements, or condensation of refractory elements (‘Which chronometers?’ section). The main uncertainty in interpretation of initial ^87^Sr/^86^Sr dates is related to identifying the events in which Rb fractionated from Sr. This could have happened during crystallisation of refractory minerals, assembly of the planetesimals and/or initial large-scale melting.

Several proxies have been used for the younger limit of accretion of igneous asteroids, although they all bear substantial uncertainties due to their model dependence and the possibility of resetting by secondary processes. Ages of igneous meteorites derived from internal isochrons approximate the time of magmatism, but this is only a loose constraint on the younger limit of accretion, since magmatism can continue for a long time after planetesimal formation. The ^182^Hf–^182^W system can date the time of metal–silicate segregation related to large-scale melting during or immediately following accretion, but this system in silicate rocks containing only small amounts of metal (such as most achondrites) is prone to impact-induced resetting [62]. Thermal models of asteroid melting by heat released by decay of ^26^Al [158] have been applied to determination of accretion ages [106], but these models critically depend on assumption of a certain initial abundance of ^26^Al, and cannot be considered reliable due to the mounting evidence of ^26^Al distribution heterogeneity. The key to achieving more reliable accretion chronology is using several independent approaches with different model assumptions together and making sure that their results are consistent.

## CONCLUSIONS

New developments in mass spectrometry and sample preparation allowed improvements in the precision of ages determined using Pb–isotope and extinct radionuclide chronometers of 0.1–0.3 Ma or better by the end of 2000s. At the same time, studies of isotope fractionation (e.g. ^238^U/^235^U) and nucleosynthetic isotope variability revealed much greater heterogeneity and complexity of the ESS materials than previously imagined. In order to attain the level of accuracy of isotopic dates that matches analytical precision, natural isotope variability has to be documented and corrected for. One of the discovered complexities—the heterogeneous distribution of ^26^Al and ^92^Nb—poses a serious challenge to extinct radionuclide chronometry. The patterns of these heterogeneities need to be established, and the distribution of other extinct radionuclides, which is now perceived as homogeneous, needs to be checked.

The sources of systematic uncertainties in isotopic ages of ESS materials, both analytical and geochemical, are being gradually documented and mitigated, but there is much work to be done. Establishing reliable time formation intervals of CAIs, chondrules and the earliest achondrites with precision better than 0.3–0.5 Ma will require dedicated studies involving multiple chronometers applied to the same best-preserved materials, with supporting in-depth petrological and geochemical information, and the framework of nucleosynthetic affinity. This should be done without sacrificing analytical throughput, in order to keep up with new findings of unique meteorites, and deliveries of extremely precious asteroidal samples by space missions.

## Supplementary Material

nwaf281_Supplemental_File

## References

[1] Dalrymple GB . The age of the Earth in the twentieth century: a problem (mostly) solved. In: Conference on Celebrating the Age of the Earth. London: The Geological Society of London, 2000, 205–21.

[2] Krot AN, Bizzarro M. Chronology of meteorites and the early solar system. Geochim Cosmochim Acta 2009; 73: 4919–21. 10.1016/j.gca.2009.04.039

[3] Davis A, McKeegan K. 1.11 Short-lived radionuclides and early solar system chronology. In: Treatise on Geochemistry, 2nd Edition. Amsterdam and Boston: Elsevier, 2014, 361–95.

[4] Wadhwa M . 1.12 Solar system time scales from long-lived radioisotopes in meteorites and planetary materials. In: Treatise on Geochemistry, 2nd Edition. Amsterdam and Boston: Elsevier, 2014, 397–418.

[5] Davis AM . Short-lived nuclides in the Early Solar System: abundances, origins, and applications. Annu Rev Nucl Part Sci 2022; 72: 339–63. 10.1146/annurev-nucl-010722-074615

[6] Anand A, Mezger K. Early solar system chronology from short-lived chronometers. Geochemistry 2023; 83: 126004. 10.1016/j.chemer.2023.126004

[7] Bouvier A, Bermingham KR, Füri E. Planetary materials: a record of early Solar System events to planetary processes. In: Anbar A, Weis D (eds.). Treatise of Geochemistry. Amsterdam and Boston: Elsevier, 2025.

[8] Amelin Y, Davis WJ. Isotopic analysis of lead in sub-nanogram quantities by TIMS using a ^202^Pb–^205^Pb spike. J Anal At Spectrom 2006; 21: 1053–61. 10.1039/B606842A

[9] Connelly JN, Bizzarro M, Thrane K et al. The Pb–Pb age of angrite SAH99555 revisited. Geochim Cosmochim Acta 2008; 72: 4813–24. 10.1016/j.gca.2008.06.007

[10] Amelin Y . U–Pb ages of angrites. Geochim Cosmochim Acta 2008; 72: 221–32. 10.1016/j.gca.2007.09.034

[11] Connelly JN, Bizzarro M, Krot AN et al. The absolute chronology and thermal processing of solids in the solar protoplanetary disk. Science 2012; 338: 651–5. 10.1126/science.1226919 23118187

[12] Zhou C, Huyskens MH, Lang X et al. Calibrating the terminations of Cryogenian global glaciations. Geology 2019; 47: 251–4. 10.1130/G45719.1

[13] Szymanowski D, Schoene B. U–Pb ID-TIMS geochronology using ATONA amplifiers. J Anal At Spectrom 2020; 35: 1207–16. 10.1039/D0JA00135J

[14] Chen JH, Wasserburg GJ. Isotopic determination of uranium in picomole and subpicomole quantities. Anal Chem 1981; 53: 2060–7. 10.1021/ac00236a027

[15] Stirling CH, Halliday AN, Porcelli D. In search of live ^247^Cm in the early solar system. Geochim Cosmochim Acta 2005; 69: 1059–71. 10.1016/j.gca.2004.06.034

[16] Stirling CH, Halliday AN, Potter E-K et al. A low initial abundance of ^247^CM in the early solar system and implications for *r*-process nucleosynthesis. Earth Planet Sci Lett 2006; 251: 386–97. 10.1016/j.epsl.2006.09.023

[17] Stirling CH, Andersen MB, Potter E-K et al. Low-temperature isotopic fractionation of uranium. Earth Planet Sci Lett 2007; 264: 208–25. 10.1016/j.epsl.2007.09.019

[18] Weyer S, Anbar AD, Gerdes A et al. Natural fractionation of ^238^U/^235^U. Geochim Cosmochim Acta 2008; 72: 345–59. 10.1016/j.gca.2007.11.012

[19] Brennecka GA, Borg LE, Hutcheon ID et al. Natural variations in uranium isotope ratios of uranium ore concentrates: understanding the ^238^U/^235^U fractionation mechanism. Earth Planet Sci Lett 2010; 291: 228–33. 10.1016/j.epsl.2010.01.023

[20] Amelin Y, Kaltenbach A, Iizuka T et al. U–Pb chronology of the Solar System's oldest solids with variable ^238^U/^235^U. Earth Planet Sci Lett 2010; 300: 343–50. 10.1016/j.epsl.2010.10.015

[21] Bouvier A, Spivak-Birndorf LJ, Brennecka GA et al. New constraints on early Solar System chronology from Al–Mg and U–Pb isotope systematics in the unique basaltic achondrite Northwest Africa 2976. Geochim Cosmochim Acta 2011; 75: 5310–23. 10.1016/j.gca.2011.06.033

[22] Brennecka GA, Wadhwa M. Uranium isotope compositions of the basaltic angrite meteorites and the chronological implications for the early Solar System. Proc Natl Acad Sci USA 2012; 109: 9299–303. 10.1073/pnas.1114043109 22647606 PMC3386092

[23] Tissot FLH, Dauphas N, Grove TL. Distinct ^238^U/^235^U ratios and REE patterns in plutonic and volcanic angrites: geochronologic implications and evidence for U isotope fractionation during magmatic processes. Geochim Cosmochim Acta 2017; 213: 593–617. 10.1016/j.gca.2017.06.045

[24] Huyskens MH, Amelin Y, Yin QZ et al. ^238^U/^235^U isotopic variations in angrites and their constituent minerals. Geochim Cosmochim Acta 2025; 399: 205–20. 10.1016/j.gca.2025.04.030

[25] Krestianinov E, Amelin Y, Yin QZ et al. Igneous meteorites suggest aluminium-26 heterogeneity in the early Solar Nebula. Nat Commun 2023; 14: 4940. 10.1038/s41467-023-40026-1 37643999 PMC10465487

[26] Bollard J, Connelly JN, Whitehouse MJ et al. Early formation of planetary building blocks inferred from Pb isotopic ages of chondrules. Sci Adv 2017; 3: e1700407. 10.1126/sciadv.1700407 28808680 PMC5550225

[27] Koefoed P, Amelin Y, Yin QZ et al. U–Pb and Al–Mg systematics of the ungrouped achondrite Northwest Africa 7325. Geochim Cosmochim Acta 2016; 183: 31–45. 10.1016/j.gca.2016.03.028

[28] Tissot FLH, Ibanez-Mejia M, Boehnke P et al. ^238^U/^235^U measurement in single-zircon crystals: implications for the Hadean environment, magmatic differentiation and geochronology. J Anal At Spectrom 2019; 34: 2035–52. 10.1039/C9JA00205G

[29] Catanzaro EJ, Murphy TJ, Shields WR et al. Absolute isotopic abundance ratios of common equal-atom and radiogenic lead isotopic standards. J Res Natl Bur Stand A 1968; 72A: 261–7. 10.6028/jres.072A.025 PMC6624684 31824095

[30] Connelly JN, Condon D. Interlaboratory calibration of mass spectrometric methods used for Pb-Pb dating of meteorites under the auspices of the EarlyTime initiative. V.M. Goldschmidt Conference, Sacramento, 8–13 June 2014.

[31] Huyskens MH, Sanborn ME, Yin Q-Z. High-precision U-Pb and Pb-Pb geochronology at UC Davis – First results for EarlyTime standards. 46th Lunar and Planetary Science Conference. Houston, 16–20 March 2015.

[32] Amelin Y, Yin QZ, Koefoed P et al. Fractionation of radiogenic Pb isotopes in meteorites and their components induced by acid leaching. Geochim Cosmochim Acta 2025; 392: 52–69. 10.1016/j.gca.2024.11.008

[33] Kita NT, Ushikubo T, Knight KB et al. Internal ^26^Al–^26^Mg isotope systematics of a type B CAI: remelting of refractory precursor solids. Geochim Cosmochim Acta 2012; 86: 37–51. 10.1016/j.gca.2012.02.015

[34] Barrat JA, Chaussidon M, Yamaguchi A et al. A 4,565-My-old andesite from an extinct chondritic protoplanet. Proc Natl Acad Sci USA 2021; 118: e2026129118. 10.1073/pnas.2026129118 33836612 PMC7980472

[35] McKibbin SJ, Ireland TR, Amelin Y et al. Mn–Cr relative sensitivity factors for secondary ion mass spectrometry analysis of Mg–Fe–Ca olivine and implications for the Mn–Cr chronology of meteorites. Geochim Cosmochim Acta 2013; 110: 216–28. 10.1016/j.gca.2013.02.025

[36] Doyle PM, Jogo K, Nagashima K et al. Mn–Cr relative sensitivity factor in ferromagnesian olivines defined for SIMS measurements with a Cameca ims-1280 ion microprobe: implications for dating secondary fayalite. Geochim Cosmochim Acta 2016; 174: 102–21. 10.1016/j.gca.2015.10.010

[37] McCain KAA, Matsuda N, Liu M-C et al. Early fluid activity on Ryugu inferred by isotopic analyses of carbonates and magnetite. Nat Astron 2023; 7: 309–17. 10.1038/s41550-022-01863-0

[38] Kawasaki N, Yamamoto D, Wada S et al. ^26^Al–^26^Mg chronology of high-temperature condensate hibonite in a fine-grained, Ca-Al-rich inclusion from reduced CV chondrite. Meteorit Planet Sci 2024; 59: 630–9. 10.1111/maps.13989

[39] Ogliore RC, Huss GR, Nagashima K. Ratio estimation in SIMS analysis. Nucl Instr Meth Phys Res B 2011; 269: 1910–8. 10.1016/j.nimb.2011.04.120

[40] Teng F-Z, Dauphas N, Watkins JM. Non-traditional stable isotopes: retrospective and prospective. In: Teng FZ, Watkins J, Dauphas N (eds.). Non-Traditional Stable Isotopes. Chantilly: Mineralogical Society of America, 2017, 1–26. 10.1515/9783110545630

[41] Di YK, Yin QZ, Tissot FLH et al. Role of natural isotopic fractionation in isotope geo- and cosmo-chronology: a theoretical investigation. Geochim Cosmochim Acta 2024; 379: 1–22. 10.1016/j.gca.2024.06.012

[42] Nyquist LE, Kleine T, Shih CY et al. The distribution of short-lived radioisotopes in the early solar system and the chronology of asteroid accretion, differentiation, and secondary mineralization. Geochim Cosmochim Acta 2009; 73: 5115–36. 10.1016/j.gca.2008.12.031

[43] Mukhopadhyay S . Early differentiation and volatile accretion recorded in deep-mantle neon and xenon. Nature 2012; 486: 101–4. 10.1038/nature11141 22678288

[44] Halliday AN, Porcelli D. In search of lost planets—the paleocosmochemistry of the inner solar system. Earth Planet Sci Lett 2001; 192: 545–59. 10.1016/S0012-821X(01)00479-4

[45] Hans U, Kleine T, Bourdon B. Rb–Sr chronology of volatile depletion in differentiated protoplanets: BABI, ADOR and ALL revisited. Earth Planet Sci Lett 2013; 374: 204–14. 10.1016/j.epsl.2013.05.029

[46] Wasserburg GJ, Papanastassiou DA, Sanz HG. Initial strontium for a chondrite and determination of a metamorphism or formation interval. Earth Planet Sci Lett 1969; 7: 33–43. 10.1016/0012-821X(69)90008-9

[47] Podosek FA, Brannon JC. Chondrite chronology by initial ^87^Sr/^86^Sr in phosphates. Meteoritics 1991; 26: 145–52. 10.1111/j.1945-5100.1991.tb01030.x

[48] Yobregat E, Fitoussi C, Bourdon B. A new method for TIMS high precision analysis of Ba and Sr isotopes for cosmochemical studies. J Anal At Spectrom 2017; 32: 1388–99. 10.1039/C7JA00012J

[49] Di YK, Krestianinov E, Zink S et al. High-precision multidynamic Sr isotope analysis using thermal ionization mass spectrometer (TIMS) with correction of fractionation drift. Chem Geol 2021; 582: 120411. 10.1016/j.chemgeo.2021.120411

[50] Begemann F, Ludwig KR, Lugmair GW et al. Call for an improved set of decay constants for geochronological use. Geochim Cosmochim Acta 2001; 65: 111–21. 10.1016/S0016-7037(00)00512-3

[51] Schiller M, Connelly JN, Glad AC et al. Early accretion of protoplanets inferred from a reduced inner solar system ^26^Al inventory. Earth Planet Sci Lett 2015; 420: 45–54. 10.1016/j.epsl.2015.03.028 27429474 PMC4946628

[52] Bollard J, Kawasaki N, Sakamoto N et al. Combined U-corrected Pb-Pb dating and ^26^Al-^26^Mg systematics of individual chondrules—evidence for a reduced initial abundance of ^26^Al amongst inner Solar System chondrules. Geochim Cosmochim Acta 2019; 260: 62–83. 10.1016/j.gca.2019.06.025

[53] Connelly JN, Bollard J, Amsellem E et al. Evidence for very early planetesimal formation and ^26^Al/^27^Al heterogeneity in the protoplanetary disk. Astrophys J Lett 2023; 952: L33. 10.3847/2041-8213/ace42e

[54] Desch SJ, Dunlap DR, Dunham ET et al. Statistical chronometry of meteorites. I. A test of ^26^Al homogeneity and the Pb-Pb age of the solar system's *t* = 0. Icarus 2023; 402: 115607. 10.1016/j.icarus.2023.115607

[55] Piralla M, Villeneuve J, Schnuriger N et al. A unified chronology of dust formation in the early solar system. Icarus 2023; 394: 115427. 10.1016/j.icarus.2023.115427

[56] Desch SJ . Extending statistical chronometry to Tafassasset, Erg Chech 002, and other Achondrites. 86th Annual Meeting of the Meteoritical Society. Brussels, July 28 to August 2 2024.

[57] Haba MK, Lai Y-J, Wotzlaw J-F et al. Precise initial abundance of niobium-92 in the Solar System and implications for *p*-process nucleosynthesis. Proc Natl Acad Sci USA 2021; 118: e2017750118. 10.1073/pnas.2017750118 33608458 PMC7923630

[58] Hibiya Y, Iizuka T, Enomoto H et al. Evidence for enrichment of niobium-92 in the outer protosolar disk. Astrophys J Lett 2023; 942: L15. 10.3847/2041-8213/acab5d

[59] Amelin Y, Koefoed P, Iizuka T et al. U-Pb, Rb-Sr and Ar-Ar systematics of the ungrouped achondrites Northwest Africa 6704 and Northwest Africa 6693. Geochim Cosmochim Acta 2019; 245: 628–42. 10.1016/j.gca.2018.09.021 PMC6457475 30983599

[60] Sanborn ME, Wimpenny J, Williams CD et al. Carbonaceous achondrites Northwest Africa 6704/6693: milestones for early Solar System chronology and genealogy. Geochim Cosmochim Acta 2019; 245: 577–96. 10.1016/j.gca.2018.10.004

[61] Mane P, Render J, Brennecka GA et al. Distribution of ^92^Nb in the early Solar system. 55th Lunar and Planetary Science Conference. Houston, 11–15 March 2024.

[62] Kleine T, Touboul M, Bourdon B et al. Hf–W chronology of the accretion and early evolution of asteroids and terrestrial planets. Geochim Cosmochim Acta 2009; 73: 5150–88. 10.1016/j.gca.2008.11.047

[63] Villa IM, De Bièvre P, Holden NE et al. IUPAC-IUGS recommendation on the half life of ^87^Rb. Geochim Cosmochim Acta 2015; 164: 382–5. 10.1016/j.gca.2015.05.025

[64] Villa IM, Bonardi ML, De Bièvre P et al. IUPAC-IUGS status report on the half-lives of ^238^U, ^235^U and ^234^U. Geochim Cosmochim Acta 2016; 172: 387–92. 10.1016/j.gca.2015.10.011

[65] Villa IM, Holden NE, Possolo A et al. IUPAC-IUGS recommendation on the half-lives of ^147^Sm and ^146^Sm. Geochim Cosmochim Acta 2020; 285: 70–7. 10.1016/j.gca.2020.06.022

[66] Heinitz S, Kajan I, Schumann D. How accurate are half-life data of long-lived radionuclides? Radiochim Acta 2022; 110: 589–608. 10.1515/ract-2021-1135

[67] Kutschera W, Billquist PJ, Frekers D et al. Half-life of ^60^Fe. Nucl Instr Meth Phys Res B 1984; 5: 430–5.

[68] Shukolyukov A, Lugmair GW. Live iron-60 in the early solar-system. Science 1993; 259: 1138–42. 10.1126/science.259.5098.1138 17794393

[69] Tachibana S, Huss GR. The initial abundance of ^60^Fe in the solar system. Astrophys J 2003; 588: L41–4. 10.1086/375362

[70] Tang H, Dauphas N. Low ^60^Fe abundance in Semarkona and Sahara 99555. Astrophys J 2015; 802: 22. 10.1088/0004-637X/802/1/22

[71] Trappitsch R, Boehnke P, Stephan T et al. New constraints on the abundance of ^60^Fe in the early solar system. Astrophys J Lett 2018; 857: L15. 10.3847/2041-8213/aabba9

[72] Kodolànyi J, Hoppe P, Vollmer C et al. Iron-60 in the early solar system revisited: insights from in situ isotope analysis of chondritic troilite. Astrophys J 2022; 929: 107. 10.3847/1538-4357/ac5910

[73] Nurmia M, Graeffe G, Valli K et al. Alpha activity of Sm-146. Ann Acad Sci Fennicae 1964; AVI: 148.

[74] Friedman A, Milsted J, Metta D et al. Alpha decay half lives of ^148^Gd ^150^Gd and ^146^Sm. Radiochim Acta 1966; 5: 192–4. 10.1524/ract.1966.5.4.192

[75] Kinoshita N, Paul M, Kashiv Y et al. RETRACTED: A shorter ^146^Sm half-life measured and implications for ^146^Sm-^142^Nd chronology in the solar system. Science 2012; 335: 1614–7. 10.1126/science.1215510 22461609

[76] Kinoshita N, Paul M, Kashiv Y et al. RETRACTION: A shorter ^146^Sm half-life measured and implications for ^146^Sm-^142^Nd chronology in the solar system. Science 2023; 379: 1307. 10.1126/science.adh7739 22461609

[77] Chiera NM, Sprung P, Amelin Y et al. The ^146^Sm half-life re-measured: consolidating the chronometer for events in the early Solar System. Sci Rep 2024; 14: 17436. 10.1038/s41598-024-64104-6 39090187 PMC11294585

[78] Kim GB, Borg LE, Boyd STP et al. Absolute decay counting of ^146^Sm and ^147^Sm for early solar system chronology. J Low Temp Phys 2022; 209: 824–31. 10.1007/s10909-022-02798-6

[79] Kavner ARL, Shollenberger QR, Kmak KN et al. Absolute decay counting of ^146^Sm with 4π cryogenic microcalorimetry. Nucl Instr Meth Phys Res A 2024; 1065: 169462. 10.1016/j.nima.2024.169462.

[80] Nakamura T, Noguchi T, Tanaka M et al. Itokawa dust particles: a direct link between S-type asteroids and ordinary chondrites. Science 2011; 333: 1113–6. 10.1126/science.1207758 21868667

[81] Yurimoto H, Abe K-I, Abe M et al. Oxygen isotopic compositions of asteroidal materials returned from Itokawa by the Hayabusa Mission. Science 2011; 333: 1116–9. 10.1126/science.1207776 21868668

[82] Yokoyama T, Nagashima K, Nakai I et al. Samples returned from the asteroid Ryugu are similar to Ivuna-type carbonaceous meteorites. Science 2023; 379: eabn7850. 10.1126/science.abn7850 35679354

[83] Yokoyama T, Wadhwa M, Iizuka T et al. Water circulation in Ryugu asteroid affected the distribution of nucleosynthetic isotope anomalies in returned sample. Sci Adv 2023; 9: eadi7048. 10.1126/sciadv.adi7048 37939187 PMC10631728

[84] Tissot FLH, Collinet M, Namur O et al. The case for the angrite parent body as the archetypal first-generation planetesimal: large, reduced and Mg-enriched. Geochim Cosmochim Acta 2022; 338: 278–301. 10.1016/j.gca.2022.09.031

[85] Keil K . Angrites, a small but diverse suite of ancient, silica-undersaturated volcanic-plutonic mafic meteorites, and the history of their parent asteroid. Geochemistry 2012; 72: 191–218. 10.1016/j.chemer.2012.06.002

[86] Jin Z, Zhang Y, Bose M et al. Petrogenesis of Erg Chech 002 achondrite and implications for an altered Magma Ocean. Astrophys J 2024; 965: 24. 10.3847/1538-4357/ad2ea7

[87] Deligny C, Piralla M, Villeneuve J et al. Potential chronological disturbance of the D'Orbigny angrite inferred from discordant ^26^Al ages. Astrophys J Lett 2024; 975: L16. 10.3847/2041-8213/ad8654

[88] Carlson RW, Boyet M, Horan M. Chondrite barium, neodymium, and samarium isotopic heterogeneity and early Earth differentiation. Science 2007; 316: 1175–8. 10.1126/science.1140189 17525335

[89] Trinquier A, Birck J-L, Allegre CJ. Widespread ^54^Cr heterogeneity in the inner solar system. Astrophys J 2007; 655: 1179–85. 10.1086/510360

[90] Trinquier A, Elliott T, Ulfbeck D et al. Origin of nucleosynthetic isotope heterogeneity in the solar protoplanetary disk. Science 2009; 324: 374–6. 10.1126/science.1168221 19372428

[91] Warren PH . Stable-isotopic anomalies and the accretionary assemblage of the Earth and Mars: a subordinate role for carbonaceous chondrites. Earth Planet Sci Lett 2011; 311: 93–100. 10.1016/j.epsl.2011.08.047

[92] Kruijer TS, Burkhardt C, Budde G et al. Age of Jupiter inferred from the distinct genetics and formation times of meteorites. Proc Nat Acad Sci USA 2017; 114: 6712–6. 10.1073/pnas.1704461114 28607079 PMC5495263

[93] Sanborn ME, Yin Q-Z. Magmatism in the Outer Solar System: what we know now from isotope forensics of carbonaceous achondrites. 50th Lunar and Planetary Science Conference. Houston, 18–22 March 2019.

[94] Kleine T, Budde G, Burkhardt C et al. The non-carbonaceous–carbonaceous meteorite dichotomy. Space Sci Rev 2020; 216: 55. 10.1007/s11214-020-00675-w

[95] Kruijer TS, Kleine T, Borg LE. The great isotopic dichotomy of the early Solar System. Nat Astron 2020; 4: 32–40. 10.1038/s41550-019-0959-9 PMC7617084 39655089

[96] Schneider JM, Burkhardt C, Kleine T. Distribution of *s*-, *r*-, and *p*-process nuclides in the early Solar System inferred from Sr isotope anomalies in meteorites. Astrophys J Lett 2023; 952: L25. 10.3847/2041-8213/ace187

[97] Yap TE, Tissot FLH. The NC-CC dichotomy explained by significant addition of CAI-like dust to the Bulk Molecular Cloud (BMC) composition. Icarus 2023; 405: 115680. 10.1016/j.icarus.2023.115680

[98] van Kooten E, Zhao X, Franchi I et al. The nucleosynthetic fingerprint of the outermost protoplanetary disk and early Solar System dynamics. Sci Adv 2024; 10: eadp1613. 10.1126/sciadv.adp1613 38875339 PMC11177941

[99] Bizzarro M, Johansen A, Dorn C. The cosmochemistry of planetary systems. Nat Rev Chem 2025; 9: 378–96. 10.1038/s41570-025-00711-9 40295893

[100] Williams CD, Sanborn ME, Defouilloy C et al. Chondrules reveal large-scale outward transport of inner Solar System materials in the protoplanetary disk. Proc Natl Acad Sci USA 2020; 117: 23426–35. 10.1073/pnas.2005235117 32900966 PMC7519341

[101] Schrader DL, Torrano ZA, Foustoukos DI et al. Reassessing the proposed “CY chondrites”: evidence for multiple meteorite types and parent bodies from Cr-Ti-H-C-N isotopes and bulk elemental compositions. Geochim Cosmochim Acta 2025; 390: 24–37. 10.1016/j.gca.2024.12.021

[102] Bermingham KR, Füri E, Lodders K et al. The NC-CC isotope dichotomy: implications for the chemical and isotopic evolution of the early Solar System. Space Sci Rev 2020; 216: 133. 10.1007/s11214-020-00748-w

[103] Pape J, Mezger K, Bouvier AS et al. Time and duration of chondrule formation: constraints from ^26^Al-^26^Mg ages of individual chondrules. Geochim Cosmochim Acta 2019; 244: 416–36. 10.1016/j.gca.2018.10.017

[104] Siron G, Fukuda K, Kimura M et al. High precision ^26^Al-^26^Mg chronology of chondrules in unequilibrated ordinary chondrites: evidence for restricted formation ages. Geochim Cosmochim Acta 2022; 324: 312–45. 10.1016/j.gca.2022.02.010 PMC8740609 35001941

[105] Fukuda K, Tenner TJ, Kimura M et al. A temporal shift of chondrule generation from the inner to outer Solar System inferred from oxygen isotopes and Al-Mg chronology of chondrules from primitive CM and CO chondrites. Geochim Cosmochim Acta 2022; 322: 194–226. 10.1016/j.gca.2021.12.027

[106] Sugiura N, Fujiya W. Correlated accretion ages and ε^54^Cr of meteorite parent bodies and the evolution of the solar nebula. Meteorit Planet Sci 2014; 49: 772–87.

[107] Sanborn ME, Carlson RW, Wadhwa M. ^147,146^Sm–^143,142^Nd, ^176^Lu–^176^Hf, and ^87^Rb–^87^Sr systematics in the angrites: implications for chronology and processes on the angrite parent body. Geochim Cosmochim Acta 2015; 171: 80–99. 10.1016/j.gca.2015.08.026

[108] Rider-Stokes BG, Greenwood RC, Anand M et al. Impact mixing among rocky planetesimals in the early Solar System from angrite oxygen isotopes. Nat Astron 2023; 7: 836–42. 10.1038/s41550-023-01968-0

[109] Rider-Stokes BG, Anand M, White LF et al. The impact history and prolonged magmatism of the angrite parent body. Meteorit Planet Sci 2024; 59: 23–39.

[110] Desch SJ, Dunlap DR, Williams CD et al. Statistical chronometry of meteorites: II. Initial abundances and homogeneity of short-lived radionuclides. Icarus 2023; 402: 115611. 10.1016/j.icarus.2023.115611

[111] Zinner E . Presolar grains. Treatise On Geochemistry, 2nd Edition. Amsterdam and Boston: Elsevier, 2014, 181–213.

[112] Heck PR, Greer J, Koop L et al. Lifetimes of interstellar dust from cosmic ray exposure ages of presolar silicon carbide. Proc Natl Acad Sci USA 2020; 117: 1884–9. 10.1073/pnas.1904573117 31932423 PMC6995017

[113] Koop L, Nakashima D, Heck PR et al. A multielement isotopic study of refractory FUN and F CAIs: mass-dependent and mass-independent isotope effects. Geochim Cosmochim Acta 2018; 221: 296–317. 10.1016/j.gca.2017.04.029

[114] Holst JC, Olsen MB, Paton C et al. ^182^Hf–^182^W age dating of a ^26^Al-poor inclusion and implications for the origin of short-lived radioisotopes in the early Solar System. Proc Natl Acad Sci USA 2013; 110: 8819–23. 10.1073/pnas.1300383110 23671077 PMC3670341

[115] Amelin Y, Williams CD, Wadhwa M. U-Th-Pb and Rb-Sr systematics of Allende FUN CAI CMS-1. 46th Lunar and Planetary Science Conference. Houston, 16–20 March 2015.

[116] Burkhardt C, Kleine T, Bourdon B et al. Hf–W mineral isochron for Ca, Al-rich inclusions: age of the solar system and the timing of core formation in planetesimals. Geochim Cosmochim Acta 2008; 72: 6177–97. 10.1016/j.gca.2008.10.023

[117] Morbidelli A, Marrocchi Y, Ali Ahmad A et al. Formation and evolution of a protoplanetary disk: combining observations, simulations, and cosmochemical constraints. Astron Astrophys 2024; 691: A147. 10.1051/0004-6361/202451388

[118] Bouvier A, Wadhwa M. The age of the Solar System redefined by the oldest Pb–Pb age of a meteoritic inclusion. Nat Geosci 2010; 3: 637–41. 10.1038/ngeo941

[119] Amelin Y, Sapah MS, Cooke I et al. U-Th-Pb systematics of CAIs from CV Chondrite Northwest Africa 4502. 44th Lunar and Planetary Science Conference. Houston, 18–22 March 2013.

[120] Cooke I, Sapah MS, Kaltenbach A et al. Uranium isotopic composition and trace element abundances of CAIs from CV Chondrite Northwest Africa 4502. 44th Lunar and Planetary Science Conference. Houston, 18–22 March 2013.

[121] Bouvier A, Brennecka GA, Wadhwa M. Absolute chronology of the first solids in the Solar System. Workshop on Formation of the First Solids in the Solar System. Houston, 7–9 November 2011.

[122] Wadhwa M, Kita NT, Nakashima D et al. High precision ^26^Al-^26^Mg systematics for an almost pristine refractory inclusion: implications for the absolute age of the solar system. 45th Lunar and Planetary Science Conference. Houston, 17–21 March 2014.

[123] Li H, Amelin Y, Tissot FLH. Revisiting the absolute chronology of calcium-aluminum-rich inclusions from CV chondrites. 55th Lunar and Planetary Science Conference. Houston, 11–15 March 2024.

[124] Kawasaki N, Wada S, Park C et al. Variations in initial ^26^Al/^27^Al ratios among fine-grained Ca-Al-rich inclusions from reduced CV chondrites. Geochim Cosmochim Acta 2020; 279: 1–15. 10.1016/j.gca.2020.03.045

[125] Weisberg MK, McCoy TJ, Krot AN. Systematics and evaluation of meteorite classification. In: Meteorites and the Early Solar System II. Arizona: University of Arizona Press, 2006, 19–52.

[126] Scott ER, Krot AN. Chondritic meteorites and the high-temperature nebular origins of their components. In: Krot AN, Scott ERD, Reipurth B (eds.). Chondrites and the Protoplanetary Disk. San Francisco: Astronomical Society of Pacific, 2005, 15–53.

[127] Krot AN, Amelin Y, Cassen P et al. Young chondrules in CB chondrites from a giant impact in the early Solar System. Nature 2005; 436: 989–92. 10.1038/nature03830 16107841

[128] Desch SJ, Morris MA, Connolly HC Jr et al. The importance of experiments: constraints on chondrule formation models. Meteorit Planet Sci 2012; 47: 1139–56.

[129] Stewart ST, Lock SJ, Carter PJ et al. Planetesimal impact vapor plumes and nebular shocks form chondritic mixtures. Planet Sci J 2025; 6: 108. 10.3847/PSJ/adbe71

[130] Yin Q-Z, Jacobsen B, Moynier F et al. Toward consistent chronology in the early solar system: high-resolution ^53^Mn-^53^Cr chronometry for chondrules. Astrophys J 2007; 662: L43–6. 10.1086/519282

[131] Bollard J, Connelly JN, Bizzarro M. Pb-Pb dating of individual chondrules from the CB_a_ chondrite Gujba: assessment of the impact plume formation model. Meteorit Planet Sci 2015; 50: 1197–216. 27429545 10.1111/maps.12461 PMC4946626

[132] Connelly JN, Bollard J, Costa MM et al. Improved methods for high-precision Pb–Pb dating of extra-terrestrial materials. J Anal At Spectrom 2021; 36: 2579–87. 10.1039/D1JA00299F

[133] Takeda H, Ishii T, Arai T et al. Mineralogy of the Asuka 87 and 88 eucrites and crustal evolution of the HED parent body. Antarct Meteor Res 1997; 10: 401–13.

[134] Scott ER, Greenwood RC, Franchi IA et al. Oxygen isotopic constraints on the origin and parent bodies of eucrites, diogenites, and howardites. Geochim Cosmochim Acta 2009; 73: 5835–53. 10.1016/j.gca.2009.06.024

[135] Nyquist LE, Reese Y, Wiesmann H et al. Fossil ^26^Al and ^53^Mn in the Asuka 881394 eucrite: evidence of the earliest crust on asteroid 4 Vesta. Earth Planet Sci Lett 2003; 214: 11–25. 10.1016/S0012-821X(03)00371-6

[136] Wadhwa M, Amelin Y, Bogdanovski O et al. Ancient relative and absolute ages for a basaltic meteorite: implications for timescales of planetesimal accretion and differentiation. Geochim Cosmochim Acta 2009; 73: 5189–201. 10.1016/j.gca.2009.04.043

[137] Wimpenny J, Sanborn ME, Koefoed P et al. Reassessing the origin and chronology of the unique achondrite Asuka 881394: implications for distribution of ^26^Al in the early Solar System. Geochim Cosmochim Acta 2019; 244: 478–501. 10.1016/j.gca.2018.10.006

[138] Fang L, Moynier F, Chaussidon M et al. The initial solar system abundance of ^60^Fe and early core formation of the first asteroids. Sci Adv 2025; 11: eadp9381. 10.1126/sciadv.adp9381 39772667 PMC11708873

[139] Reger PM, Roebbert Y, Neumann W et al. Al–Mg and U–Pb chronological records of Erg Chech 002 ungrouped achondrite meteorite. Geochim Cosmochim Acta 2023; 343: 33–48. 10.1016/j.gca.2022.12.025

[140] Vaci Z, Jones CM, Fike DA et al. Aluminium-magnesium systematics of evolved ungrouped achondrites. 55th Lunar and Planetary Science Conference. Houston, 11–15 March 2024.

[141] Fang LR, Frossard P, Boyet M et al. Half-life and initial Solar System abundance of ^146^Sm determined from the oldest andesitic meteorite. Proc Natl Acad Sci USA 2022; 119: e2120933119. 10.1073/pnas.2120933119 35290127 PMC8944250

[142] Yang B, Pang R, Wang Q et al. Highly heterogeneous parent body of the rare andesitic Erg Chech 002 meteorite revealed by the revisited Mn–Cr isotopic systematics. Planet Sci J 2025; 6: 73. 10.3847/PSJ/ada769

[143] Wasserburg GJ, Tera F, Papanastassiou DA et al. Isotopic and chemical investigations on Angra dos Reis. Earth Planet Sci Lett 1977; 35: 294–316. 10.1016/0012-821X(77)90133-9

[144] Lugmair GW, Galer SJG. Age and isotopic relationships among the angrites Lewis Cliff 86010 and Angra dos Reis. Geochim Cosmochim Acta 1992; 56: 1673–94. 10.1016/0016-7037(92)90234-A

[145] Baker J, Bizzarro M, Wittig N et al. Early planetesimal melting from an age of 4.5662 Gyr for differentiated meteorites. Nature 2005; 436: 1127–31. 10.1038/nature03882 16121173

[146] Amelin Y . The U–Pb systematics of angrite Sahara 99555. Geochim Cosmochim Acta 2008; 72: 4874–85. 10.1016/j.gca.2008.07.008

[147] Glavin DP, Kubny A, Jagoutz E et al. Mn-Cr isotope systematics of the D'Orbigny angrite. Meteorit Planet Sci 2004; 39: 693–700.

[148] Kleine T, Hans U, Irving AJ et al. Chronology of the angrite parent body and implications for core formation in protoplanets. Geochim Cosmochim Acta 2012; 84: 186–203. 10.1016/j.gca.2012.01.032

[149] Datta C, Amelin Y, Krestianinov E et al. Disparate Pb-isotopic ages of silicate and phosphate minerals in the diabasic angrite Northwest Africa 12320. Icarus 2024; 412: 115979. 10.1016/j.icarus.2024.115979

[150] Yamaguchi A, Clayton RN, Mayeda TK et al. A new source of basaltic meteorites inferred from Northwest Africa 011. Science 2002; 296: 334–6. 10.1126/science.1069408 11951042

[151] Floss C, Taylor LA, Promprated P et al. Northwest Africa 011: a “eucritic” basalt from a non-eucrite parent body. Meteorit Planet Sci 2005; 40: 343–60.

[152] Amelin Y, Rydeblad E, Krestianinov E et al. Pb-isotopic and initial Sr ages of the achondrite NWA 4587. 50th Lunar and Planetary Science Conference. Houston, 18–22 March 2019.

[153] Huyskens MH, Sanborn ME, Yin Q-Z et al. Chronology of carbonaceous achondrites from the Outer Solar System. 50th Lunar and Planetary Science Conference. Houston, 18–22 March 2019.

[154] Warren PH, Rubin AE, Isa J et al. Northwest Africa 6693: a new type of FeO-rich, low-Δ^17^O, poikilitic cumulate achondrite. Geochim Cosmochim Acta 2013; 107: 135–54. 10.1016/j.gca.2012.12.025

[155] Hibiya Y, Archer GJ, Tanaka R et al. The origin of the unique achondrite Northwest Africa 6704: constraints from petrology, chemistry and Re-Os, O and Ti isotope systematics. Geochim Cosmochim Acta 2019; 245: 597–627. 10.1016/j.gca.2018.04.031 30983599 PMC6457475

[156] Dunlap DR, Wadhwa M, Krestianinov E et al. Chronology of the eucrite Northwest Africa 8661: a record of ancient volcanism on Vesta. 50th Lunar and Planetary Science Conference. Houston, 18–22 March 2019.

[157] Nakamura T, Matsumoto M, Amano K et al. Formation and evolution of carbonaceous asteroid Ryugu: direct evidence from returned samples. Science 2023; 379: eabn8671. 10.1126/science.abn8671 36137011

[158] Hevey PJ, Sanders IS. A model for planetesimal meltdown by ^26^Al and its implications for meteorite parent bodies. Meteorit Planet Sci 2006; 41: 95–106.

[159] Chen JH, Wasserburg GJ. The isotopic composition of uranium and lead in Allende inclusions and meteoritic phosphates. Earth Planet Sci Lett 1981; 52: 1–15. 10.1016/0012-821X(81)90202-8

[160] Rugel G, Faestermann T, Knie K et al. New measurement of the ^60^Fe half-life. Phys Rev Lett 2009; 103: 072502. 10.1103/PhysRevLett.103.072502 19792637

[161] Wallner A, Bichler M, Buczak K et al. Settling the half-life of ^60^Fe: fundamental for a versatile astrophysical chronometer. Phys Rev Lett 2015; 114: 041101. 10.1103/PhysRevLett.114.041101 25679883

[162] Ostdiek K, Anderson T, Bauder W et al. Towards a measurement of the half-life of ^60^Fe for stellar and early Solar System models. Nucl Instr Meth Phys Res B 2015; 361: 638–42.

[163] Wing J, Swartz B, Huizenga J. New hafnium isotope, Hf^182^. Phys Rev 1961; 123: 1354. 10.1103/PhysRev.123.1354

[164] Vockenhuber C, Oberli F, Bichler M et al. New half-life measurement of ^182^Hf: improved chronometer for the early solar system. Phys Rev Lett 2004; 93: 172501. 10.1103/PhysRevLett.93.172501 15525068

[165] Paul M, Ahmad I, Kutschera W. Half-life of ^41^Ca. Zeitschrift für Physik A Hadrons and Nuclei 1991; 340: 249–54. 10.1007/BF01294672

[166] Klein J, Fink D, Middleton R et al. Determination of the half-life of ^41^Ca from measurements of Antarctic meteorites. Earth Planet Sci Lett 1991; 103: 79–83. 10.1016/0012-821X(91)90151-7

[167] Jörg G, Amelin Y, Kossert K et al. Precise and direct determination of the half-life of ^41^Ca. Geochim Cosmochim Acta 2012; 88: 51–65.

[168] Meissner F, Schmidt-Ott W-D, Ziegeler L. Half-life and α-ray energy of ^146^Sm. Zeitschrift für Physik A Atomic Nuclei 1987; 327: 171–4. 10.1007/BF01292406

[169] Greenwood RC, Burbine TH, Franchi IA. Linking asteroids and meteorites to the primordial planetesimal population. Geochim Cosmochim Acta 2020; 277: 377–406. 10.1016/j.gca.2020.02.004

[170] Greenwood RC, Burbine TH, Miller MF et al. Melting and differentiation of early-formed asteroids: the perspective from high precision oxygen isotope studies. Geochemistry 2017; 77: 1–43. 10.1016/j.chemer.2016.09.005

[171] Amelin Y, Yi KU. Th and Pb distribution between minerals in Achondrites. 55th Lunar and Planetary Science Conference. Houston, 11–15 March 2024.

[172] Jacobsen B, Yin QZ, Moynier F et al. ^26^Al–^26^Mg and ^207^Pb–^206^Pb systematics of Allende CAIs: canonical solar initial ^26^Al/^27^Al ratio reinstated. Earth Planet Sci Lett 2008; 272: 353–64. 10.1016/j.epsl.2008.05.003

